# Intelligent Photovoltaic Systems by Combining the Improved Perturbation Method of Observation and Sun Location Tracking

**DOI:** 10.1371/journal.pone.0156858

**Published:** 2016-06-21

**Authors:** Yajie Wang, Yunbo Shi, Xiaoyu Yu, Yongjie Liu

**Affiliations:** The Higher Educational Key Laboratory for Measuring & Control Technology and Instrumentations of Heilongjiang Province, School of Measurement–Control Technology and Communications Engineering, Harbin University of Science and Technology, Harbin, China; Chongqing University, CHINA

## Abstract

Currently, tracking in photovoltaic (PV) systems suffers from some problems such as high energy consumption, poor anti-interference performance, and large tracking errors. This paper presents a solar PV tracking system on the basis of an improved perturbation and observation method, which maximizes photoelectric conversion efficiency. According to the projection principle, we design a sensor module with a light-intensity-detection module for environmental light-intensity measurement. The effect of environmental factors on the system operation is reduced, and intelligent identification of the weather is realized. This system adopts the discrete-type tracking method to reduce power consumption. A mechanical structure with a level-pitch double-degree-of-freedom is designed, and attitude correction is performed by closed-loop control. A worm-and-gear mechanism is added, and the reliability, stability, and precision of the system are improved. Finally, the perturbation and observation method designed and improved by this study was tested by simulated experiments. The experiments verified that the photoelectric sensor resolution can reach 0.344°, the tracking error is less than 2.5°, the largest improvement in the charge efficiency can reach 44.5%, and the system steadily and reliably works.

## Introduction

Following the development in the world economy, energy consumption has continuously increased. The burning of fossil fuels causes serious pollution to the environment. As a clean energy, solar energy has experienced increased adoption owing to its immense radiation quantity, wide coverage area, and inexhaustible reserves. Therefore, solar photovoltaic (PV) power generation systems have become an inevitable direction in energy development for the future [1–6]. At present, the orientation of solar panels in most solar PV power generation systems is fixed, which leads to low generation efficiency. Solar tracking systems can ensure that solar panels are always oriented toward the sun, which guarantees full photoelectric conversion efficiency. In recent studies, sun-tracking methods have been mainly using photoelectric and are day-numbered [7, 8]. To track the sunlight, a photoelectric tracking system uses photosensitive sensors that can measure the sun angle. Although such a system has high sensitivity, it also has dead zones. It is easily affected by the weather and can lose the target or even track a wrong one [7]. By calculating the movement path of the sun, the day-numbered trajectory tracking method can normally work under clouds. However, its tracking precision is low, and its error accumulates. The tracking trajectory needs to be recalculated for different regions. Unable to recognize outside light intensity, this system wastes energy by continuously tracking in low or no light [8]. One of the key technonlgies accelerating the growth of solar energy sources is the power management strategy of the electrochemical energy storage system such as the Li-ion battery pack, supercapacitor pack, and dual buffer [9–13].

To make the PV cells produce more electricity, recent research has focused on maximum power point tracking (MPPT) technology. The use of the MPPT technology makes the utilization ratio of PV cells higher, and the output reaches a maximum [14–17]. At present, the main methods to realize the MPPT technology include the perturbation and observation method and the constant-voltage tracking method [18, 19]. The perturbation and observation method compares the current system output with the previous output to determine the next action and thus realizes MPPT. However, it is subject to tracking the step length. It easily oscillates near the maximum power point. In addition, it cannot distinguish the output variation caused by environmental factors, causing the output to deviate from the maximum power point [20]. The constant-voltage tracking method is an approximate tracking method, which fixes the maximum power point as a constant. This method cannot adjust the output along with the change in conditions, leading to low efficiency and large error [21].

The technical contribution of this study is an intelligent control technology for light perception and MPPT. We design and construct an intelligent tracking solar PV power generation system. The core processor of this system is a field-programmable gate array (FPGA). It uses a two-degree-of-freedom (2-DOF) mechanical system and corrects its attitude using closed-loop control. Light-searching sensors are developed using the principle of projection in which a light-intensity sensor unit is combined, making an intelligent light-search perception module. This paper proposes an effective solution to differentiate perturbations, which can avoid oscillation near the maximum power point of the perturbation observation method and malfunction under light-intensity variations. It also maximizes the conversion efficiency of solar PV cells.

## System Structure

### Overall system structure

**Fig 1:**
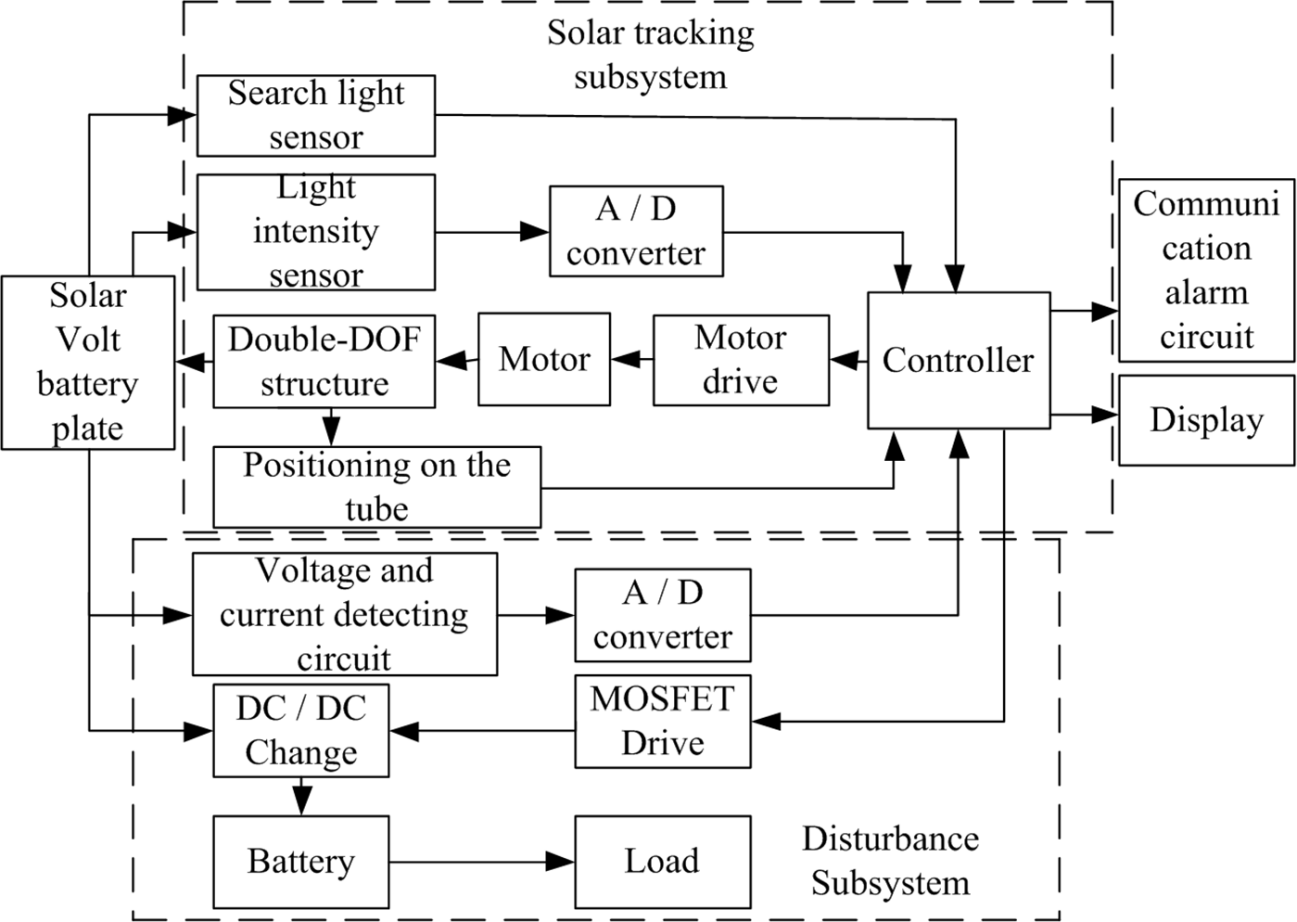
Overall system structure.

The proposed system is primarily composed of an FPGA core controller, a system power supply unit, a MOSFET drive module, a DC/DC conversion unit, a voltage/current detection module, a sun-positioning recognition module, a liquid crystal display (LCD), a motor drive module, a positioning module, and a mechanical structure. The system general structure is shown in Fig 1.

### Mechanical structure

**Fig 2:**
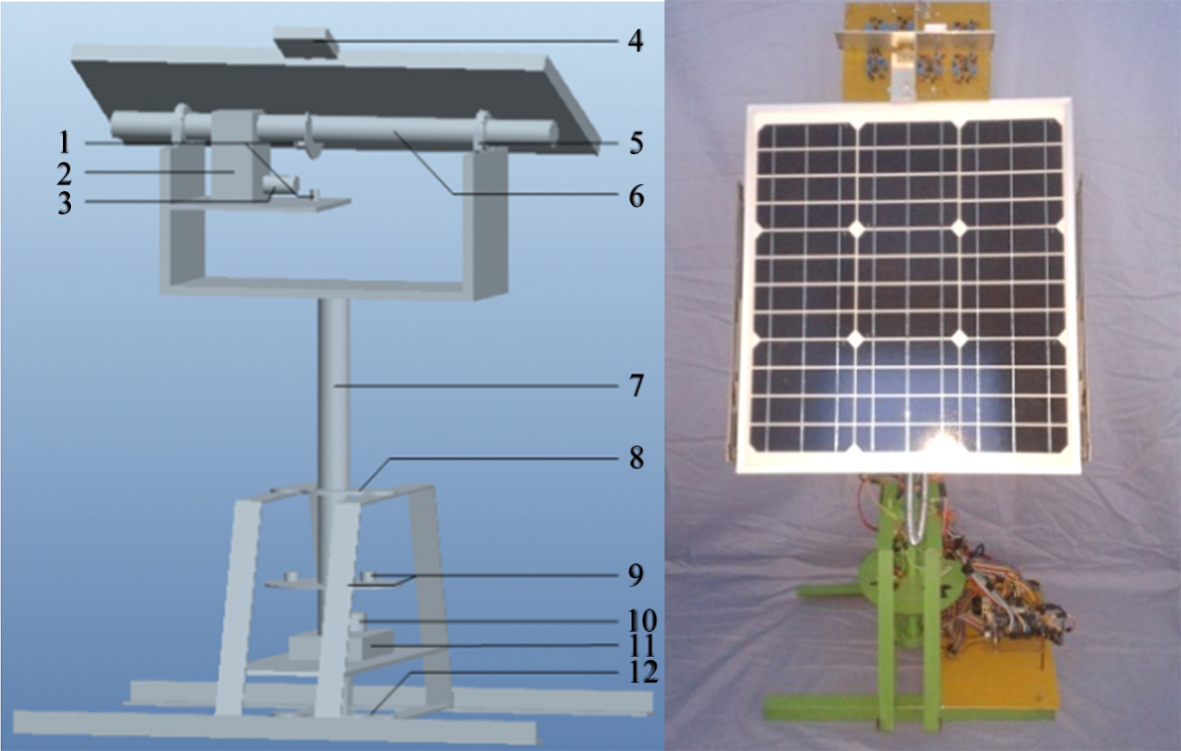
Mechanical structure. (a) Diagram of the structure. (b) Actual structure.

A level-pitch 2-DOF mechanical structure is designed to track the sun, as shown in Fig 2. Items 1 and 9 in the figure are the positioning laser geminate transistors. Item 4 is the photoelectric detection module whose main structure is a light-tracing sensor and whose top is a light-intensity-detection unit. The worm-and-gear structures (Items 2 and 11) are added to the base, which is a DC geared motor structure, to prevent reversal and overturning and to counter any driving moment caused by external stress. In this manner, the problem of counter driving moment is solved, and the control precision and stability of the system are strengthened.

### System operation

The system operation can be divided into three main parts: motor, clock, and MPPT controllers. The clock is the main component in the system operation. The system only tracks during a set period of time. During this time, the system corrects its attitude at set intervals. If vision is lost (due to environmental factors such as overcast and rainy weather), it will make a fuzzy adjustment according to the current time, which is the first interval when the environmental light intensity exceeds a photosensitive threshold, and it will find a precise position after it recovers the vision. In the set period, the system adopts a discrete-type tracking method. The system peripherals, which include the motor, motor drive module, light-tracing sensor module, positioning geminate-transistor module, light-intensity-detection module, and display module, are only charged when a set clock signal appears, and the power is interrupted and maintained in standby mode after the required detection and control. Every evening, the system attitude returns to zero, and the control part stays in the standby mode with low energy consumption. To prolong the battery service life, the system adopts a three-stage charging, which uses the MPPT technology during the large-current charging phase to rapidly provide power and to perform real-time monitoring of the environmental light intensity and battery power. During the small-current and trickle-charging stage, all equipment except the controller runs in a time-discrete mode.

## Disturbance Subsystem

### MPPT model

According to the relationgs with the output current and voltage of solar photovoltaic cells, can obtain the relations with the output voltage and power, as shown in Eq 1.

$$P=I_{ph}U-I_{0}U\left[\text{exp}(qU/nkT)-1\right]$$ (1)

where *q* is the quantity of electric charge, *n* is the diode ideal constant, *k* is Boltzmann constant (1.38×10–23) and *T* is absolute temperature(°C).

**Fig 3:**
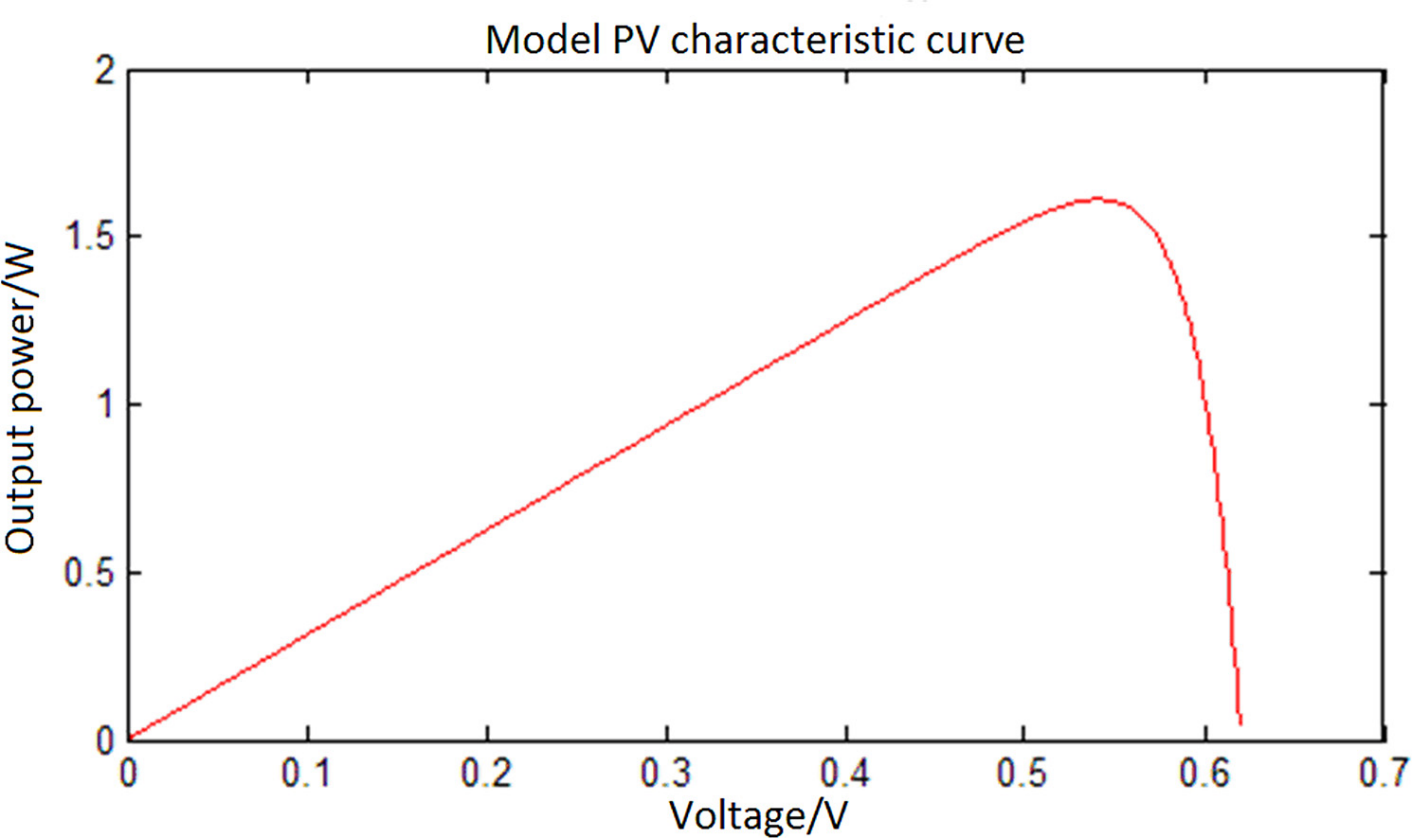
Characteristic P–V curve of PV cells.

Fig 3 shows that a PV battery can reach maximum output power by regulating the external load in a set external environment. As the output power reaches its peak, the output voltage is defined as maximum power point voltage *U*_*m*_, the electric current is defined as maximum power point current *I*_*m*_, and the output power is defined as maximum power point *P*_*m*._ Because of the influence of external factors such as temperature and light intensity, the maximum power point moves when external conditions change. Thus, a technique is needed to find the maximum power point and to obtain the maximum power output using a control algorithm, namely, the MPPT technology [22].

### Determination of the MPPT method

**Fig 4:**
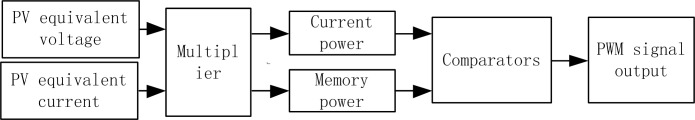
Basic schematic of the MPPT.

Realization of MPPT is a dynamic optimization process. The system controller monitors and controls the power through real-time monitoring and by regulating the output voltage and current strategy of the solar cell. To ensure that the PV cells are dynamically working at the maximum power point, the present output power should be compared with the previously measured output power, which is memorized in the controller. Then, the interference should be adjusted to increase the output power. The cycle is then repeated. MPPT is implemented as shown in Fig 4.

### Disturbance observation

**Fig 5:**
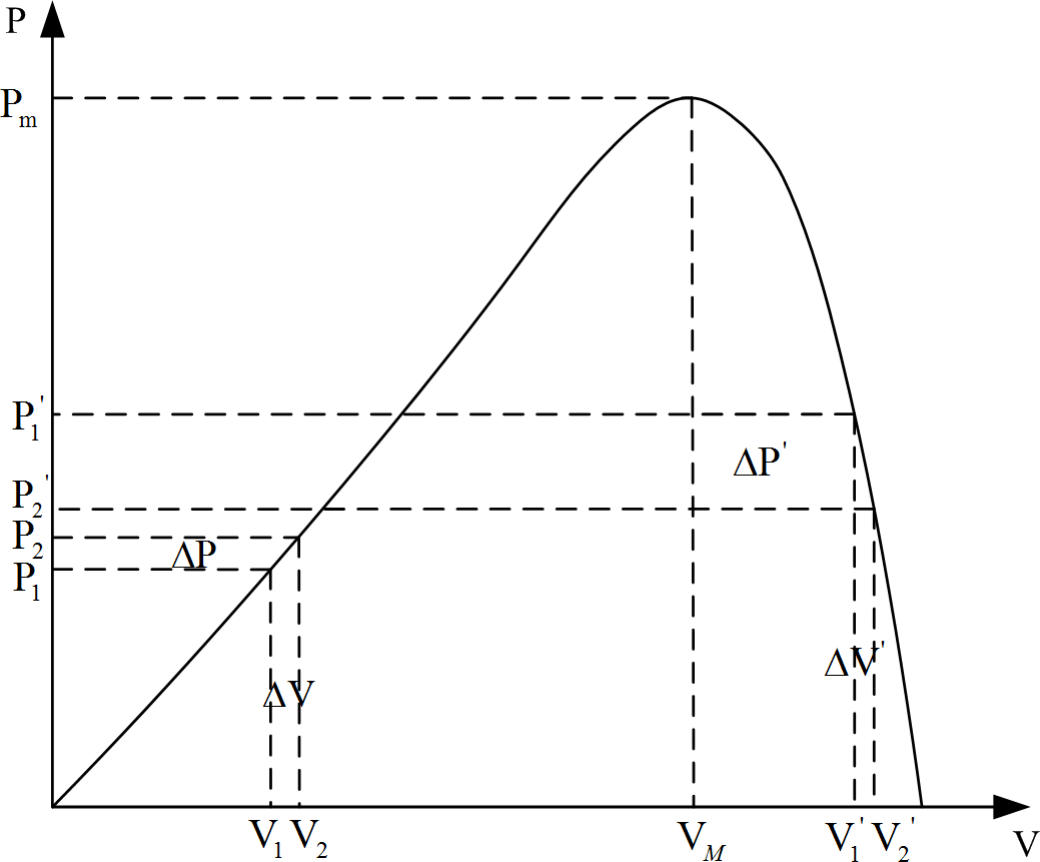
MPPT scheme.

This design incorporates a disturbance observation method to realize the MPPT algorithm. The principle is shown in Fig 5. The basic principle is to disturb the output voltage of the solar panels using pulse-width modulated (PWM) signals output by the software via the MOSFET driver circuit to continuously monitor the output power and voltage of the PV cells and to compute power variation Δ*P* and voltage variation Δ*V*. If Δ*P* is positive, the current disturbance causes the output power of the PV cells to move in the direction of maximum power point, which means that the disturbance is in the right direction. If Δ*P* is negative, the current disturbance causes the PV cell output power to decrease, which means a change in the disturbance direction is needed and so on until Δ*P* tends to zero. At this time, the system operates at the maximum power point and maintains the current duty ratio.

### Improved algorithm model

**Fig 6:**
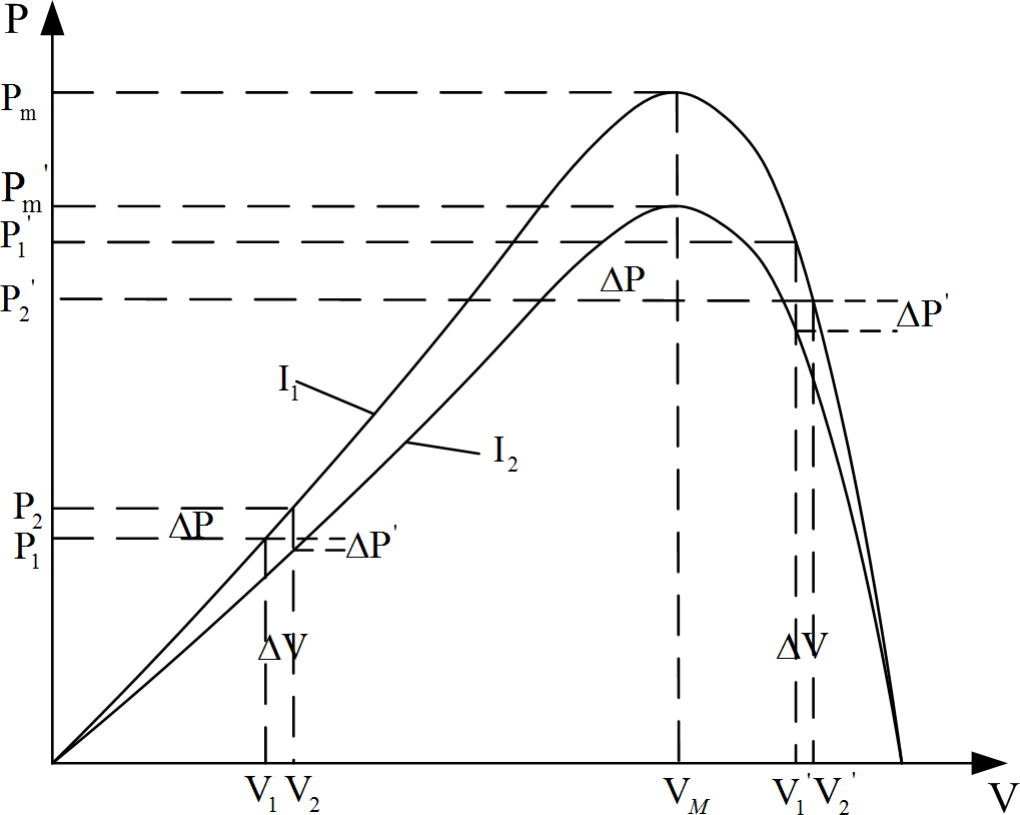
Improved disturbance observation principle.

The disturbance observation method suffers from defects such as poor anti-interference and a tendency to oscillate near the maximum power point. To solve these problems, this paper proposes an improved disturbance observation method. Fig 6 shows that when the current output is to the left of the maximum power point, the next-state Δ*P* is negative because decreasing sunshine strength causes the current power to decrease from P1 to P2. According to the discriminant method of perturbation observation, the controller reduces the output voltage *V* of the PV cells. However, when the maximum power point is to the right of the current power output, the correct adjustment method increases *V*. The same error also exists when the current power output is to the right of the maximum power point. At this moment, the disturbance observation control would perform a wrong operation in response to the illumination change.

To overcome this shortcoming, we improve the algorithm in this study. The environmental light intensity, monitored as Δ*P*, is classified as positive or negative. When Δ*P* is less than zero, the variation in the environmental light Δ*S* is also less than zero, which means that the environmental light intensity interferes with the algorithm. When the duty ratio is kept unchanged, if Δ*S* is nonnegative (which means that the ambient light does not affect the algorithm), the direction of the disturbance needs to be changed. Similarly, when both Δ*P* and Δ*S* is positive, the light-intensity change interferes with the algorithm, and the output voltage maintains its original value. Otherwise, the disturbance should continue. The improved disturbance observation also considers the influence of Δ*S* on Δ*P*. It suppresses the interference from the light-intensity change and makes up for the defect in algorithm miscalculation caused by the light-intensity changes. Because the adjusted step length of the disturbance observation method is fixed, the output power characteristic of the PV cells is not linear. Therefore, when the disturbance observation reaches the maximum power point, it would overshoot and reverberate in steady state.

## Hardware Circuit Design of the Disturbance System

### DC/DC conversion circuit

**Fig 7:**
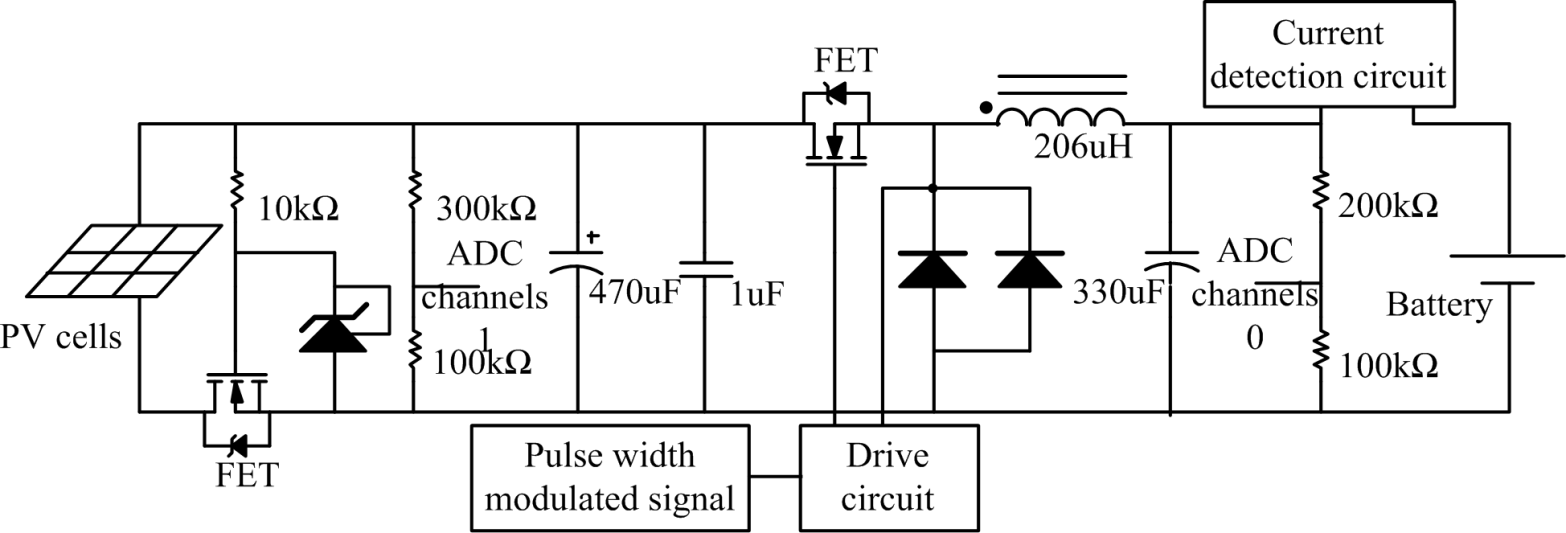
DC/DC conversion circuit.

A DC/DC conversion circuit is designed to work together with the MPPT algorithm (to make the output voltage of the PV battery controllable) and to complete the step-charging function of the battery. The DC/DC conversion circuit is directly connected to the two modules, PV cells, and battery. Therefore, security must be integrated into the circuit to make the system safe by preventing component damage caused by problems such as wrong operation and connection error. The DC/DC conversion circuit is shown in Fig 7.

The basic parameters of the DC/DC circuit are designed as follows:

1. The input voltage range is determined by the output characteristics of the PV cells; the value ranges from 13.6 to 21.9 V.
2. The maximum dropout voltage value is 200 mV (the output current ranges from 100 mA to 2.5 A within 1 ms).
3. The maximum output current is 2.5 A.
4. The switching frequency is 100 kHz.

Electrical components parameters of the DC/DC circuit were calculated as follows:

The duty ratio ranges from two ratio figures, namely, the ratio of the minimum output to the maximum input and the ratio of the maximum output to the minimum input.

$$D_{\text{min}}=\frac{V_{out,\;\text{min}}}{V_{in,\;\text{max}}}=\frac{13.6}{21.9}=0.621$$ (2)

$$D_{\text{max}}=\frac{V_{out,\;\text{max}}}{V_{in,\;\text{min}}}=\frac{13.6}{13.6}=1$$ (3)

The inductance (*L*) is chosen according to the principle of maximum voltage difference value and minimum duty ratio.

$$u=L\frac{di}{dt}$$

$$L=\frac{\left(V_{in,\;\text{max}}-V_{out}\right)\times T_{on}}{\delta \times I_{out,\;\text{max}}}$$ (4)

where the compensating coefficient value of δ is 0.1 and *T*_on_ is the PWM conduction time. Inductance *L* can be obtained as

$$L=\frac{(21.9-13.6)\times 6.21\times 10^{-6}}{0.1\times 2.5}=206.172\mu \text{H}.$$

Choosing the capacitance value should meet the condition of 250 mV (100 mA–2.5 A). The calculation expression is

$$i=C\frac{du}{dt}.$$ (5)

Eq 5 can be expressed in vector form as

$$\overset{•}{I}=j\omega C\overset{•}{U}.$$ (6)

Eq 6 leads to the capacitance expression

$$C=\frac{\overset{•}{I}}{j\omega \overset{•}{U}}$$ (7)

in which the bandwidth of crossover frequency *f*_*c*_ is obtained as

$$f_{c}=\frac{\Delta I_{out}}{2\pi C_{out}\Delta V_{out}}$$ (8)

where Δ*I*_*out*_ is the down current, Δ*V*_*out*_ is the dropout voltage, and *f*_*c*_ is the crossover frequency.

When the voltage is 10 kHz, the impedance value of the capacitance is obtained by

$$Z_{c}=\frac{\Delta V_{out}}{\Delta I_{out}}=\frac{250\text{mV}}{2.4\text{A}}=104.2\text{m}\Omega .$$ (9)

According to the calculation results, in the ZL system of RUBYCON Company, we can obtain the 16-V, 330-μF capacitance parameters as follows:

$$\begin{array}{l} I_{c,\;rms}=760\text{mA}@T_{A}=105{}^{\circ}\text{C}\\ R_{\text{ESR},\;\text{low}}=72\text{m}\Omega @T_{A}=20{}^{\circ}\text{C}\\ R_{\text{ESR},\;\text{low}}=220\text{m}\Omega @T_{A}=-10{}^{\circ}\text{C}. \end{array}$$

The capacitive impedance of the equivalent series resistance (ESR) is obtained by

$$\begin{array}{l} \frac{1}{2\pi f_{c}C_{out}}=\frac{1}{6.28\times 10k\times 330}=48\text{m}\Omega \\ Zc\leq \sqrt{R^{2}{}_{ESR}+{\left(\frac{1}{2\pi f_{c}C_{out}}\right)}^{2}}=\sqrt{72^{2}+48^{2}}=86\text{m}\Omega . \end{array}$$ (10)

The calculation results of the capacitor ESR show that the capacitive impedance is smaller than Zc, which meets the design requirements.

### MOSFET driver circuit

**Fig 8:**
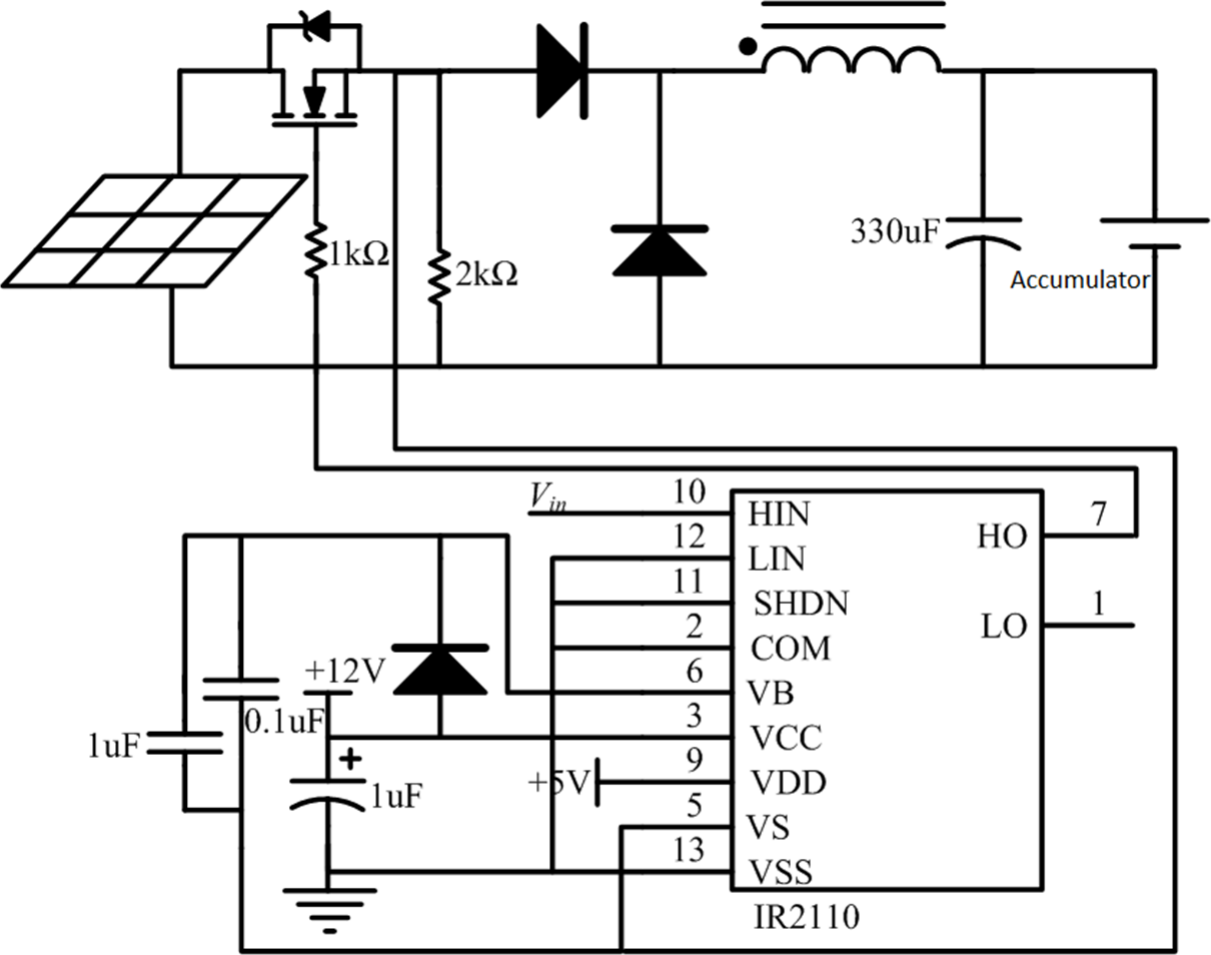
MOSFET typical driving circuit.

To realize MOSFET switch control, this design uses the IR2110 driver chip made by IR Company (U.S.). This chip, which has the advantages of both light coupling and electromagnetic separation, is the first choice of the driver circuit required in this design. A typical driving circuit is shown in Fig 8.

**Fig 9:**
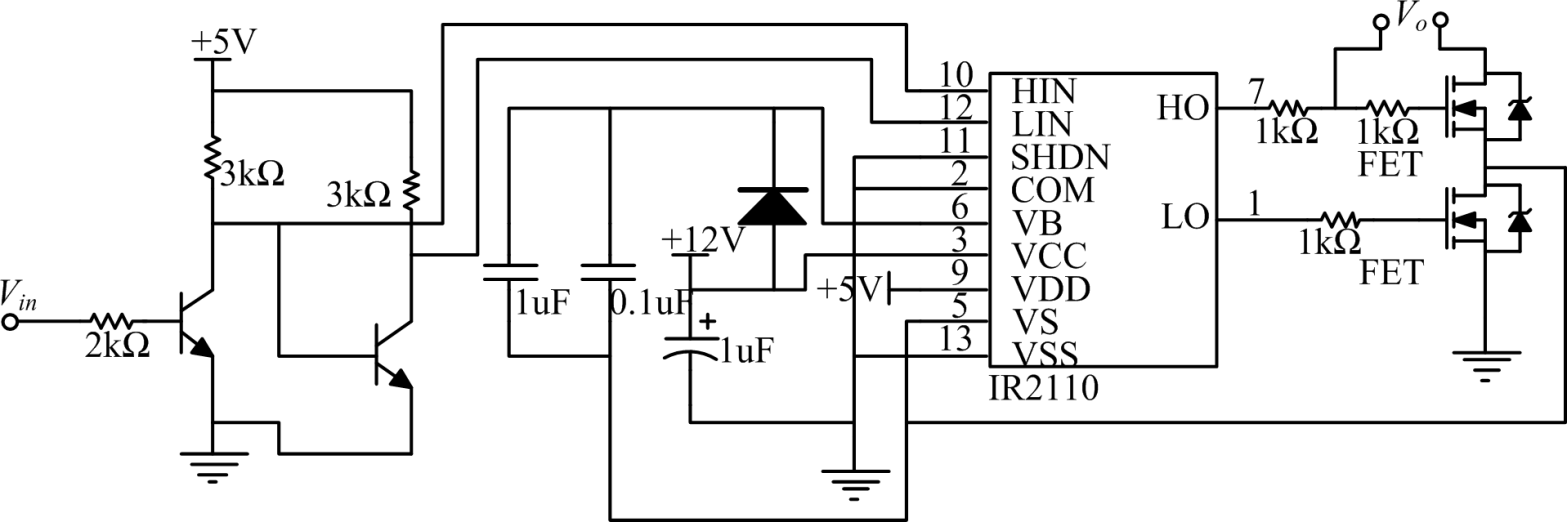
Improved MOSFET drive circuit.

To improve the performance of the driving circuit, the design in this study made some improvements to the traditional driving circuit of the IR2110, such as adopting a high–low end simultaneous reverse-switching mode, decreasing the charge and discharge times, and making the edge of the drive pulse steeper. Such improvements greatly reduced the problem of MOSFET output jitter in the threshold voltage and improved the switching speed and frequency. The improved driving circuit significantly increased the controllable range of the output voltage. After eliminating the jitter, the output voltage value was substantially the same as the theoretical value obtained by the duty-ratio calculation. The improved IR2110 drive circuit is shown in Fig 9, where HO and LO are the high and low drive pulse output ends, respectively, and HIN and LIN are the high and low logic level input ends, respectively.

The improved driving circuit expends less power than the typical one. Compared with the traditional drive circuit, the improved drive circuit eliminates the diode in the DC/DC conversion circuit (which is designed to prevent reverse signal flow) and the resistance for charging. The energy expended by the diode and the resistance cannot be ignored, especially that of the diode. Its energy dissipation is expressed as

$$P=\frac{U^{2}{}_{R}}{R}+U_{d}I_{L}=1.212\text{W}$$ (11)

where the voltage drop of the diode conduction *U*_*d*_ = 0.7 V, *R* = 2 kΩ, load average voltage *U*_*L*_ = 14 V, and average current *I*_*L*_ = 1.5 A. The voltage of the electric resistance is obtained from the equation *U*_*R*_ = *U*_*L*_*/D*, where *D* is the duty ratio, which is equal to 18 V. The improved field-effect tube increases the low energy consumption on the order of milliwatts, which can be ignored. Therefore, the improved circuit can increase the efficiency of the drive circuit in a better manner.

### A/D conversion circuit

**Fig 10:**
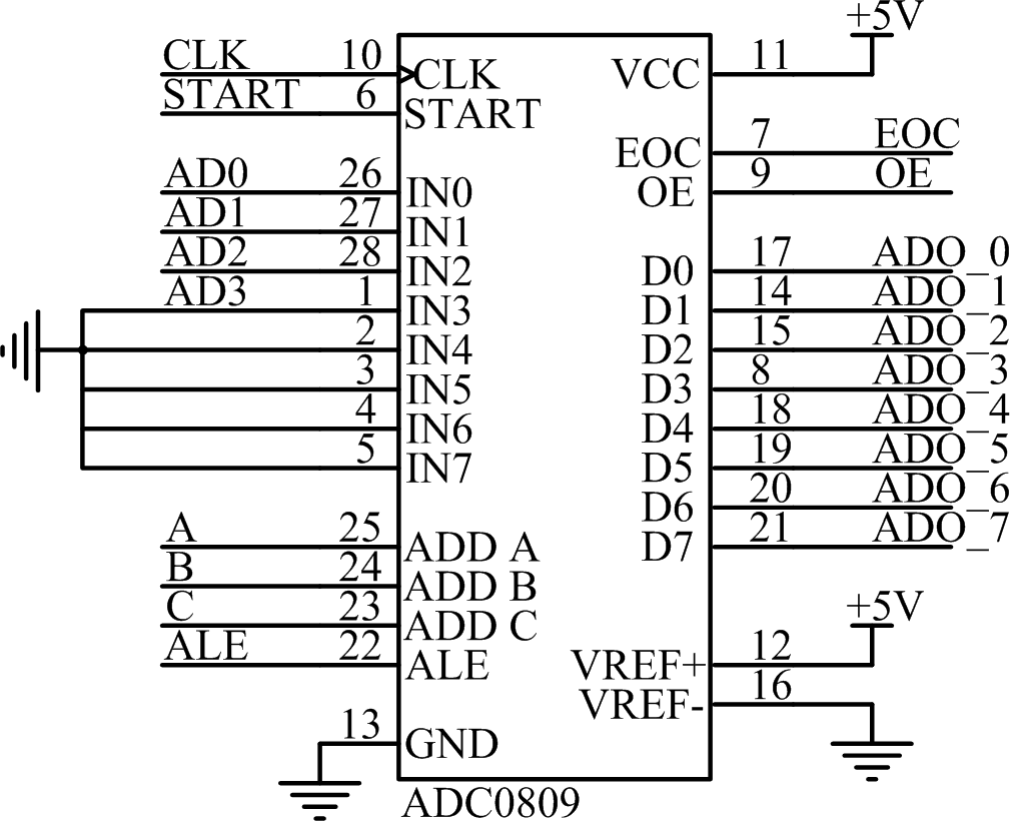
A/D converter circuit.

This design incorporates the ADC0809 eight-bit analog/digital (A/D) conversion chip as a modular transition device. The chip has eight input channels. Switching between each channel is realized by an analog switch. The analog signal input in each channel can be successively converted through time-division multiplex access. Because the A/D converter maximum input voltage is limited to a standard voltage, the standard voltage of this design is 5 V, and the highest voltage converted is 21.9 V. As a result, the measured voltage must be processed into a voltage that can be identified by the A/D converter. To be collected by the A/D converter, the measured voltage is proportionally decreased to less than 5 V by adopting a resistance divider. The A/D conversion circuit is shown in Fig 10.

### Current detection circuit

**Fig 11:**
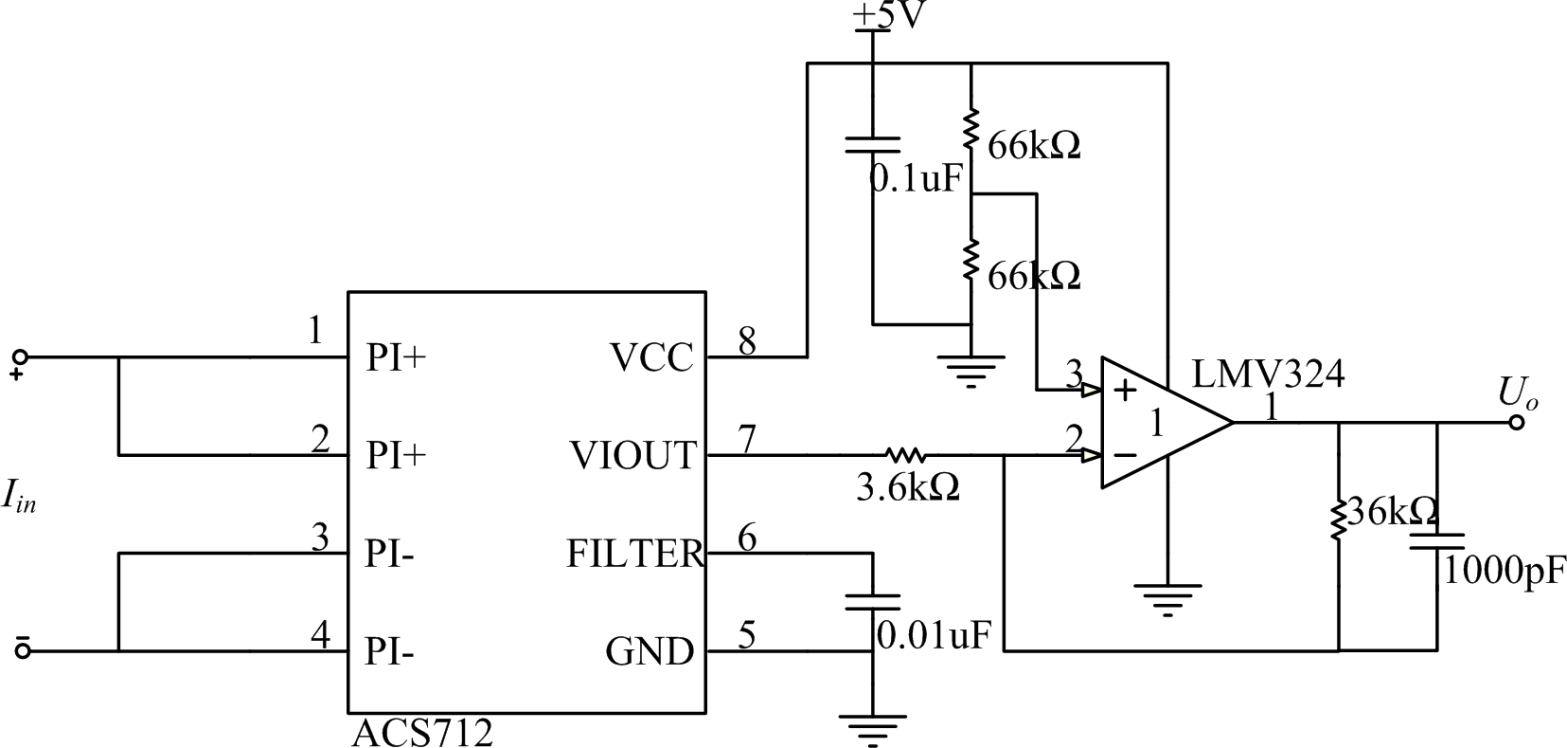
Current detection circuit.

The ACS712 Hall current sensor from Allegro Application Company is used for the current examination. Using a bidirectional DC linear output, it has the characteristics of high sensitivity, high precision, and high cost performance. The ACS712-5 type sensor is used in this design, whose detection ranges from −5 to +5 A. Its accuracy is ±1.5%, the sensitivity is 180–190 mV/A, the output reference voltage is 2.5 V, and the working temperature is 40–85°C. The detection circuit is shown in Fig 11.

Fig 11 shows that the amplifying circuit should be benchmarked against the 2.5-V standard. Because the design demand of the maximum electric current peak is 2 A, the output should be amplified by a factor of five for an output voltage range from 0.65 to 4.35 V, which can easily be collected by the A/D converter. In the diagram, PI+ and PI− are the input and output ports of the detected current, respectively. The analog signal output port is VIOUT.

### Other hardware circuit designs

The other hardware circuit designs include the communication alarm circuit, display circuit module, and voltage conversion module.

The communication and alarm circuit uses the general packet radio service (GPRS) communication methods, which apply the SIM900A module GPRS communication solutions. The 1602 LCD is chosen as the display module for real-time display of information such as the time and battery status. To meet the system power supply of each unit and the needs of the different modules on the power supply voltage, a system power supply circuit is designed and constructed. A 12-V battery is used as the main battery for the supply current. It supplies power to the motor drive module, display module, light-searching sensors, laser tube positioning, and realization of the MPPT circuit and FPGA minimum system using voltage-conversion chips 7808, 2940, and 1117–3.3–5.0.

## Software Design

### System software general structure design

The following introduces the overall design of the software system:

Main working procedure of the system:

1. Initializing the program
2. Calculating the sun trajectory and determining the light intensity and battery level
3. Simultaneously controlling the first attitude and operating the MPPT
4. Connecting the GPRS network
5. Running the system

**Fig 12:**
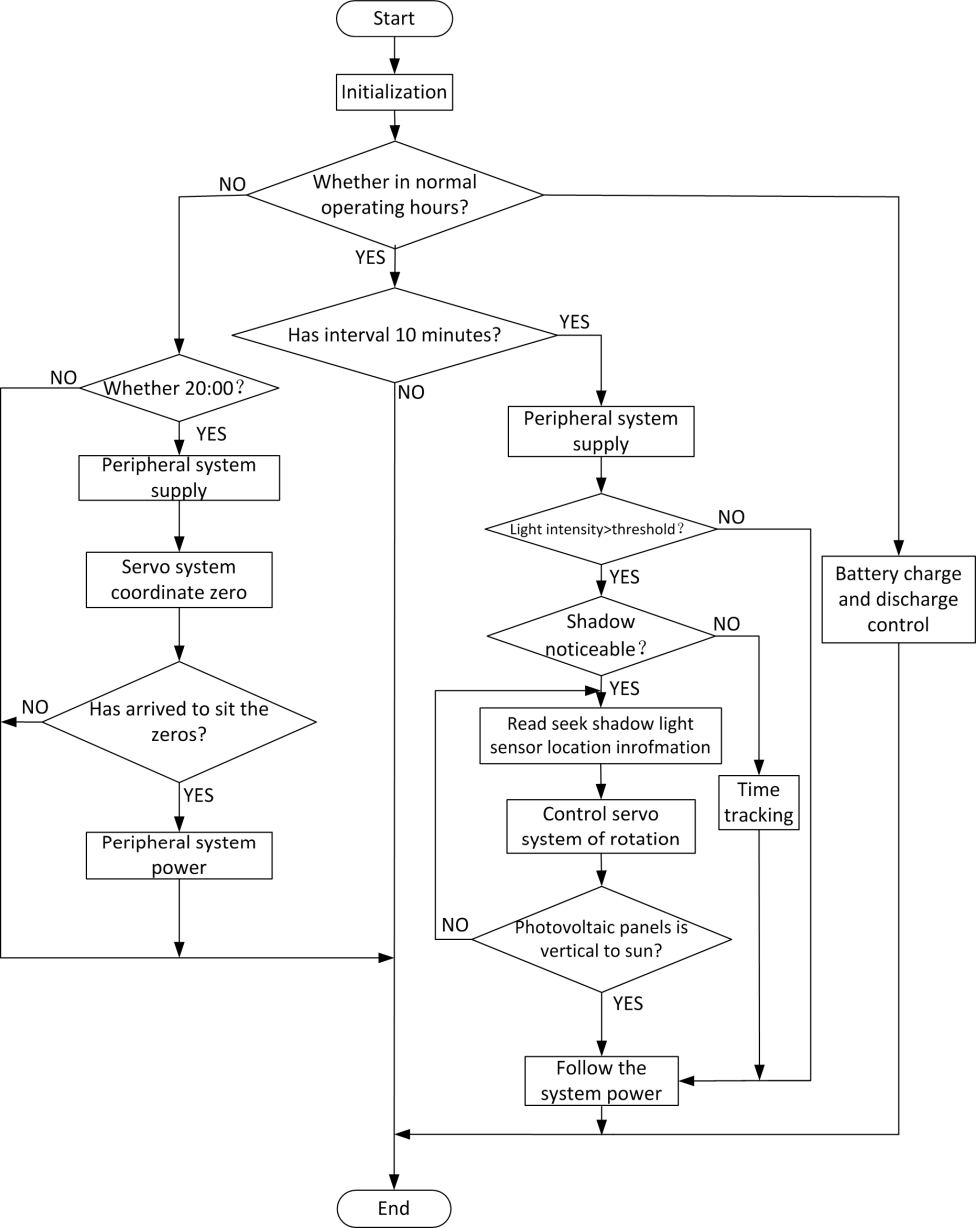
Program flow of the system software.

At the very start, the program initializes the processor registers and the system clock and sets the system performance period, standby period, discrete time interval, and the MPPT algorithm parameters. Next, the program initializes the A/D converter and GPRS, calculates the sun trajectory, and completes the determination of the current light intensity and battery level. The program roughly adjusts the sun tracking once more, precisely adjusts the position system attitude by photoelectric tracking, completes the first attitude adjustment, and runs the MPPT. While running, the system uses a discrete type of solar tracking with a tracking interval of 10 min, i.e., the system adjusts its attitude at set intervals. In other periods, each module of the tracking subsystem interrupts the power supply and remains in standby. During the running time, if the sun position identification sensor loses the position due to weather factors (such as rainy or cloudy conditions), the program would deal with the situation according to the change in the current light intensity. If the light intensity is higher than the sensory threshold value, the trajectory tracking methods will be chosen. If the light intensity is lower than the threshold power, the system will remain in standby. The charging mode of the system is three-phase charging, and MPPT is used in the large-current charging phase. The communication and alarm circuit varies the current-running state. It transmits an alarm when an exception occurs. The system software process is shown in Fig 12.

### Software implementation process of the improved disturbance observation method

**Fig 13:**
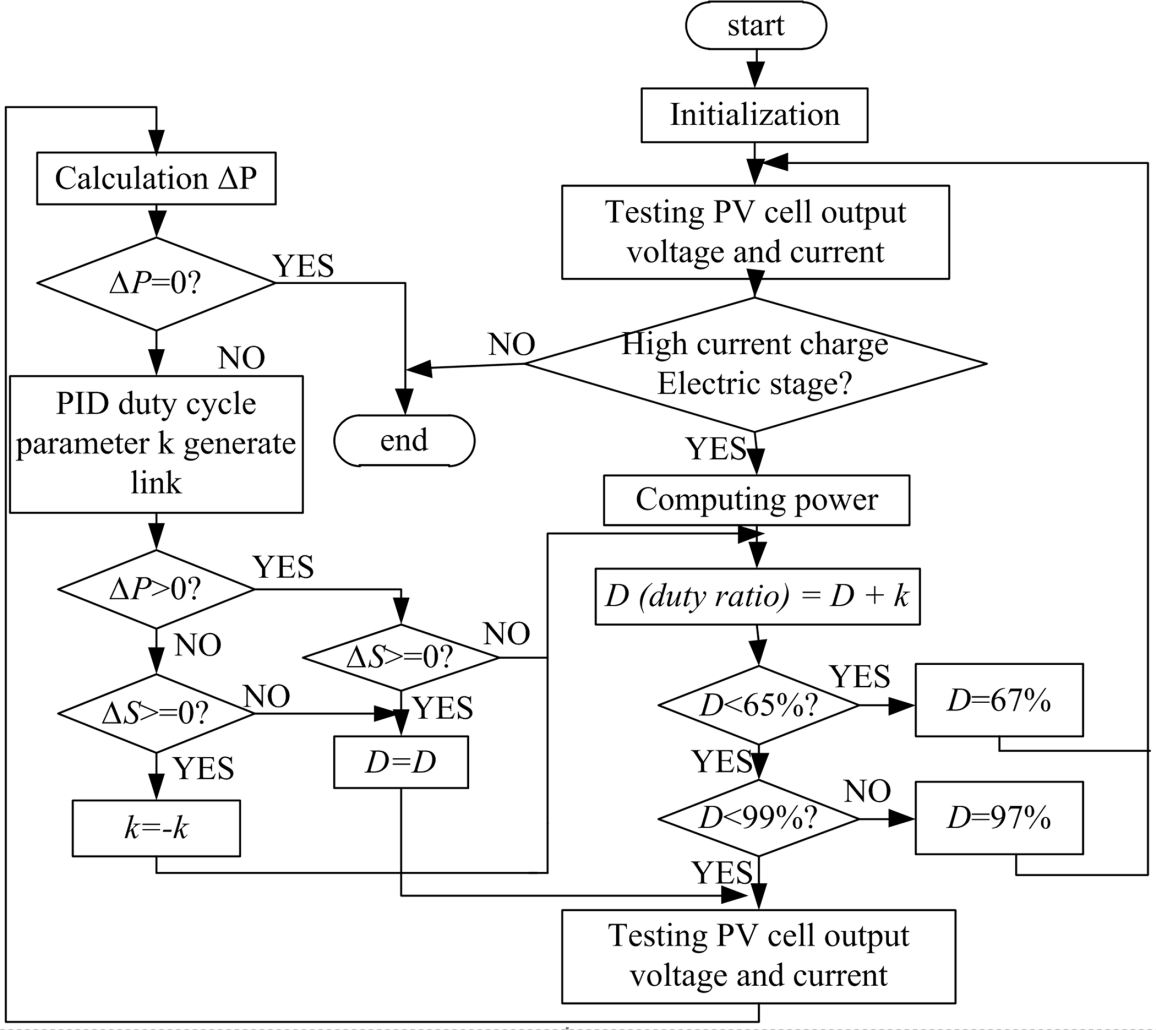
Software flowchart of the improved disturbance observation.

The software process of the improved disturbance observation method is shown in Fig 13. After initialization, the program first scans the battery level. Tracking of the maximum power point begins when the charging phase is identified as large-current charging. In the MPPT process, strong ambient light *S* is monitored in real time, and its effect on the algorithm is eliminated. Meanwhile, the scope of the duty cycle is delimited. When the duty-ratio adjustment reaches the limit of the duty-ratio boundaries, the system judgment is considered incorrect, and the duty ratio will be reset to return the system to its normal state. Proportional–integral–derivative (PID) control is used in the entire tracking progress. It adjusts the tracking step length, makes the output more stable, and avoids shock.

### Duty-ratio-generated subroutines

**Fig 14:**
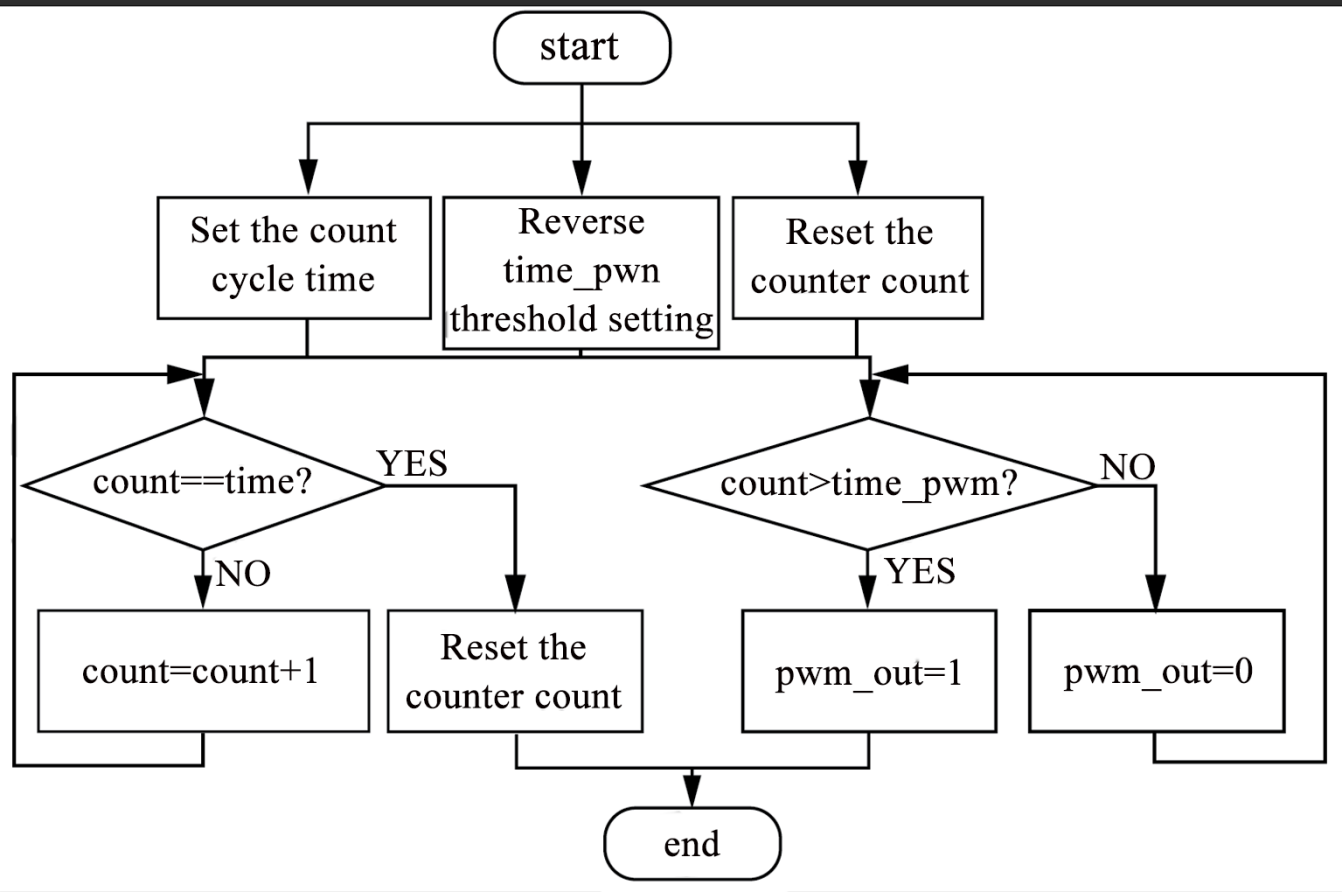
Duty cycle-generation software processes.

Duty-ratio-generated subroutines are used to transfer the duty-ratio values coming from the main program into the hardware-identifiable PWM signal. The duty-ratio-generated subroutine process is shown in Fig 14, which shows that the PWM signal cycle is determined by “time.” The actual cycle is the product of the crystal vibration cycle and “time.” The pulse width is determined by the reverse threshold “time PWM.” The actual pulse width is the product of “time PWM” and crystal vibration cycle.

### A/D conversion subroutine

**Fig 15:**
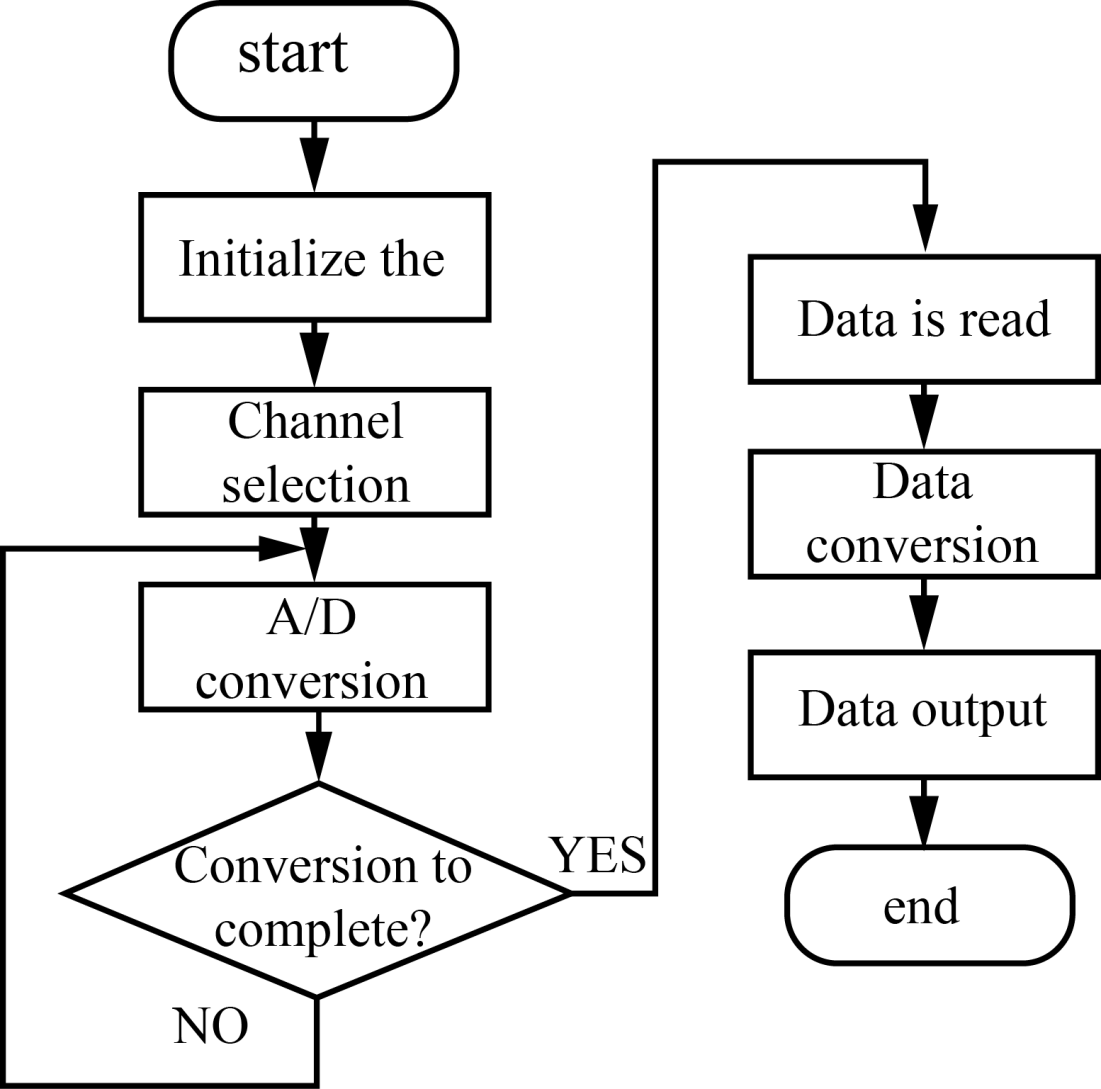
Program flow of the A/D conversion.

The A/D converter is used to complete the module conversion function. The program first collects the output voltage of the PV cells, battery terminal voltage, and current through channel selection. Then, it proportionately restores the data coming from the A/D conversion into the corresponding voltage and current values. Finally, these data are output and used by the main program. The A/D conversion program is shown in Fig 15.

## Model Simulation and System Test

### MATLAB/Simulink simulation model of perturbation observation

**Fig 16:**
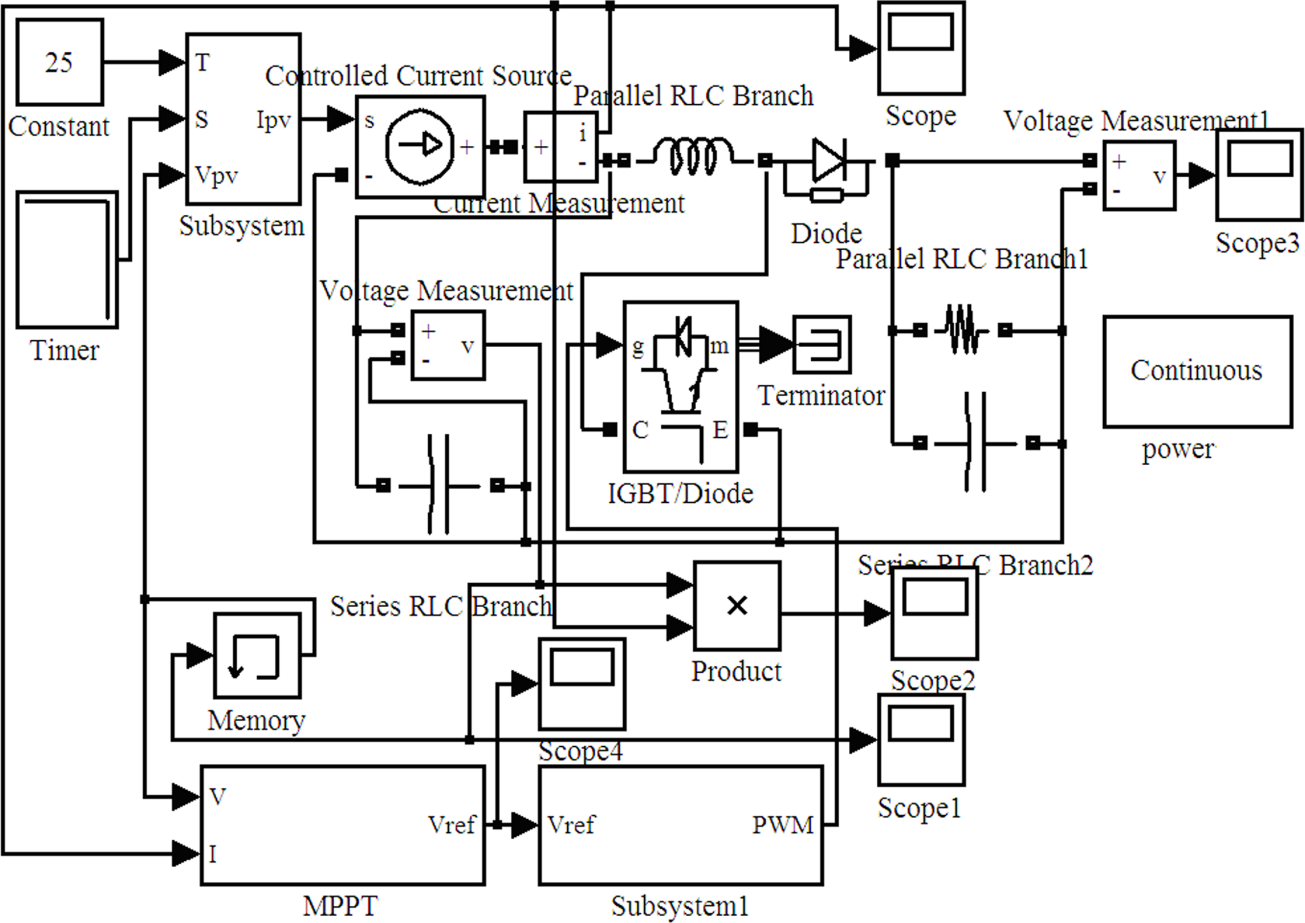
Disturbance observation of the MPPT Simulink model.

The MPPT simulation model is composed of the PV cell output current *I*_*pv*_ model, the MPPT model, which realizes the disturbance observation; the PWM model, which transfers the duty-ratio data into a PWM signal; and the basic circuit link modules. These components must be modeled in the Simulink environment. Each simulation model is modeled according to the equivalent mathematical model of each module. Fig 16 shows the basic mathematical model of the disturbance observation method to realize MPPT in which input parameters *T*, *S*, and *Vpv* are the environment temperature, light intensity, and output voltage of the PV cells, respectively. *Ipv* is the actual model current of the PV cells.

**Fig 17:**
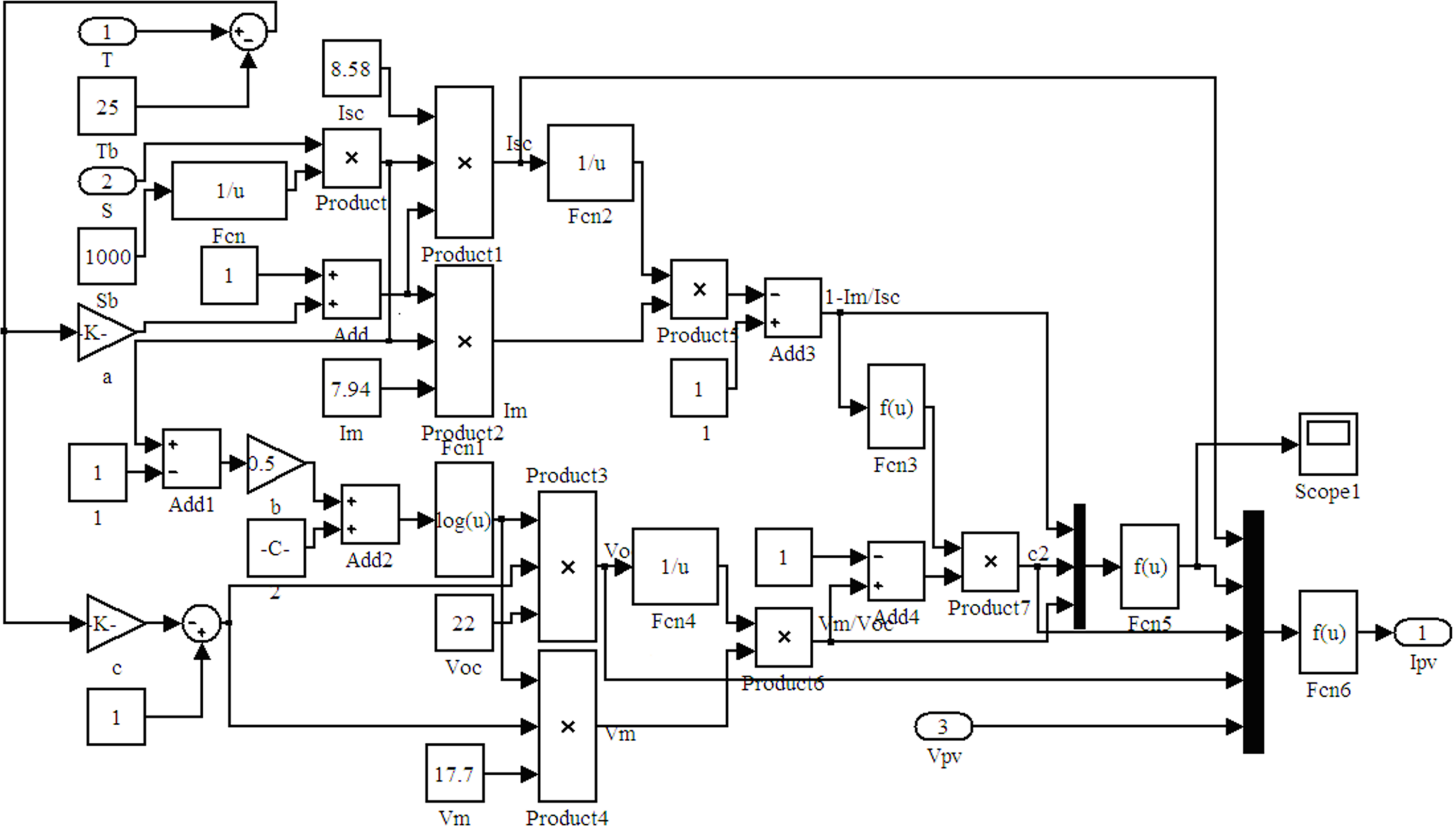
PV output current simulation model.

The PV cell output current (*Ipv*) model is determined by formula (1). Its Simulink simulation mathematical model is shown in Fig 17. *T*, *S*, and *Vpv* are quantities of the input control. *Ipv* is the output.

**Fig 18:**
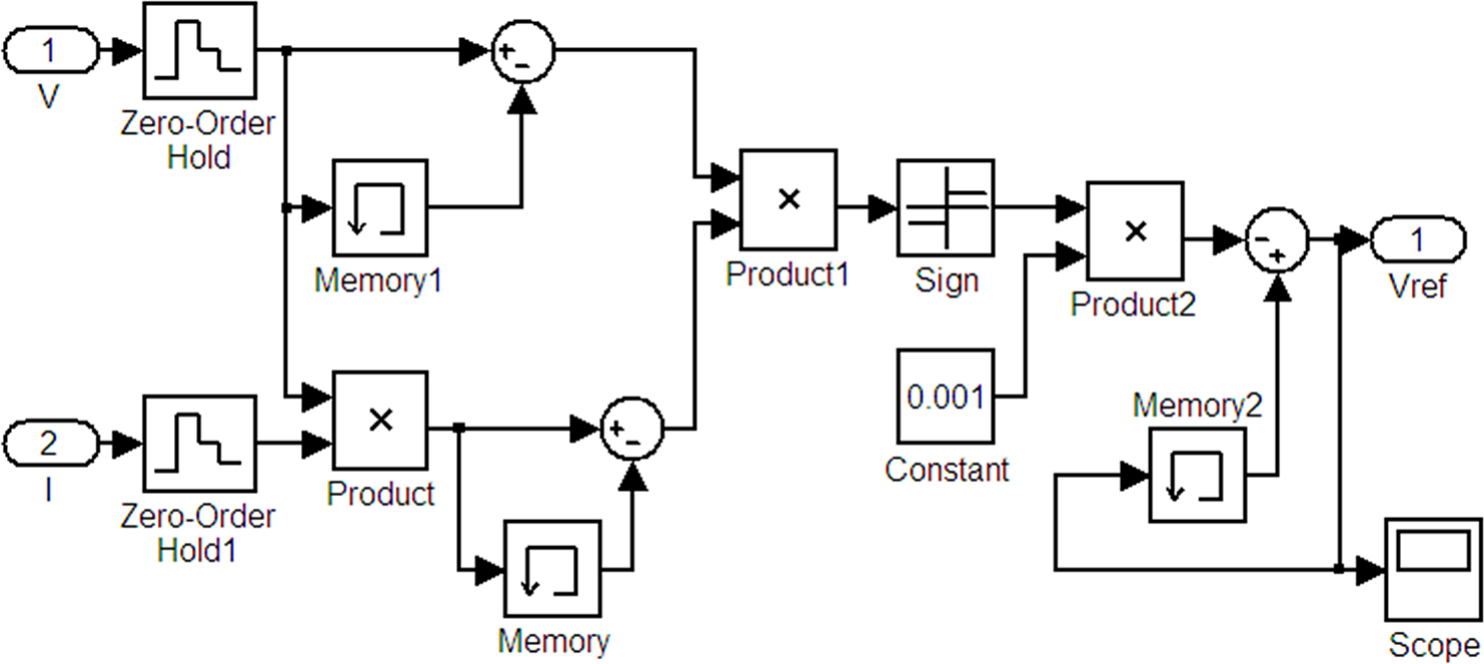
Model of the MPPT disturbance observation.

To realize the MPPT model of the disturbance observation, the current power calculation must be completed. Two values are needed: the difference with the previous state voltage and the difference with the previous state voltage. In addition, the duty-ratio adjustment direction should be determined. Finally, the adjusted duty-ratio value is determined. The Simulink model is shown in Fig 18. *V* and *I* are the input control quantity; *Vref* is the output.

**Fig 19:**
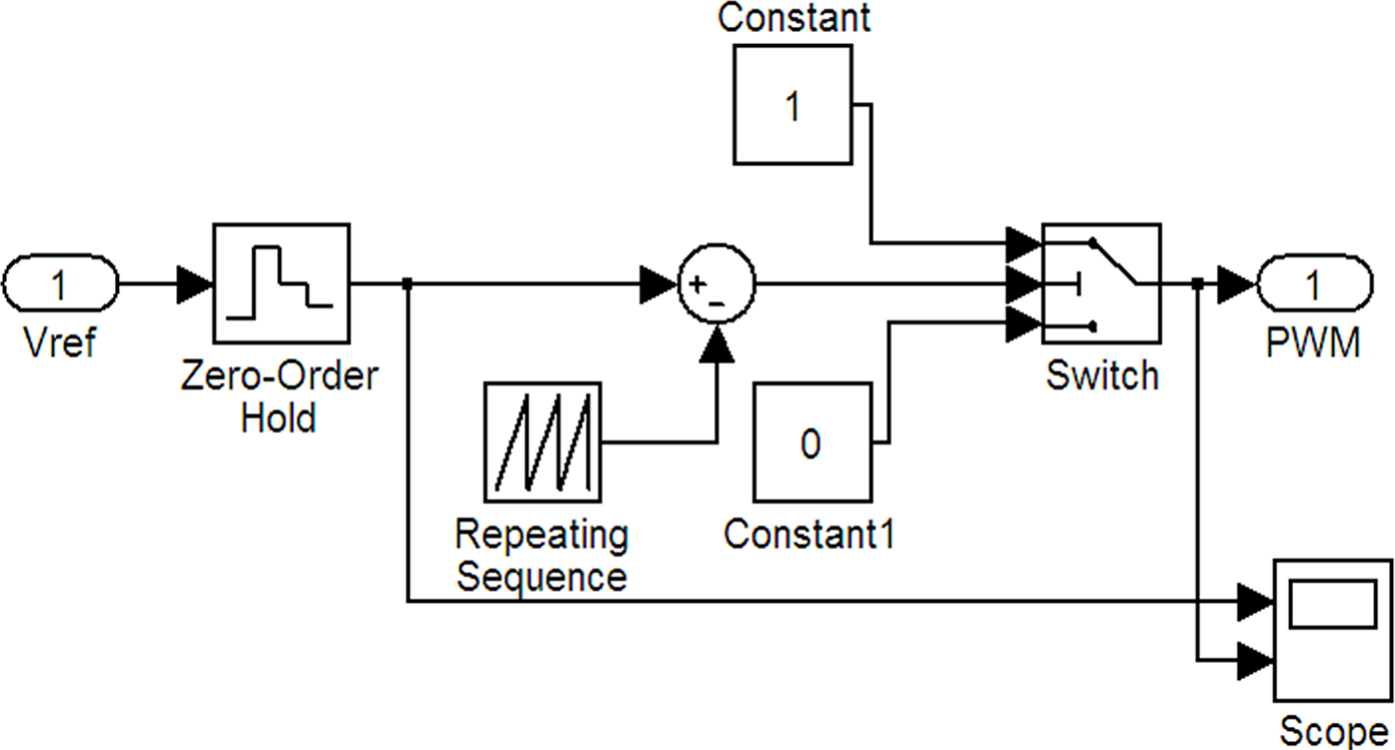
PWM generation model.

Fig 19 shows the PWM model that converts the duty-ratio data into a PWM signal. Its input variable *Vref* is the duty ratio. PWM represents the output PWM signal. The model is used to transfer the input duty ratio to the corresponding PWM signal output.

### Analysis of the disturbance observation method simulation results

**Fig 20:**
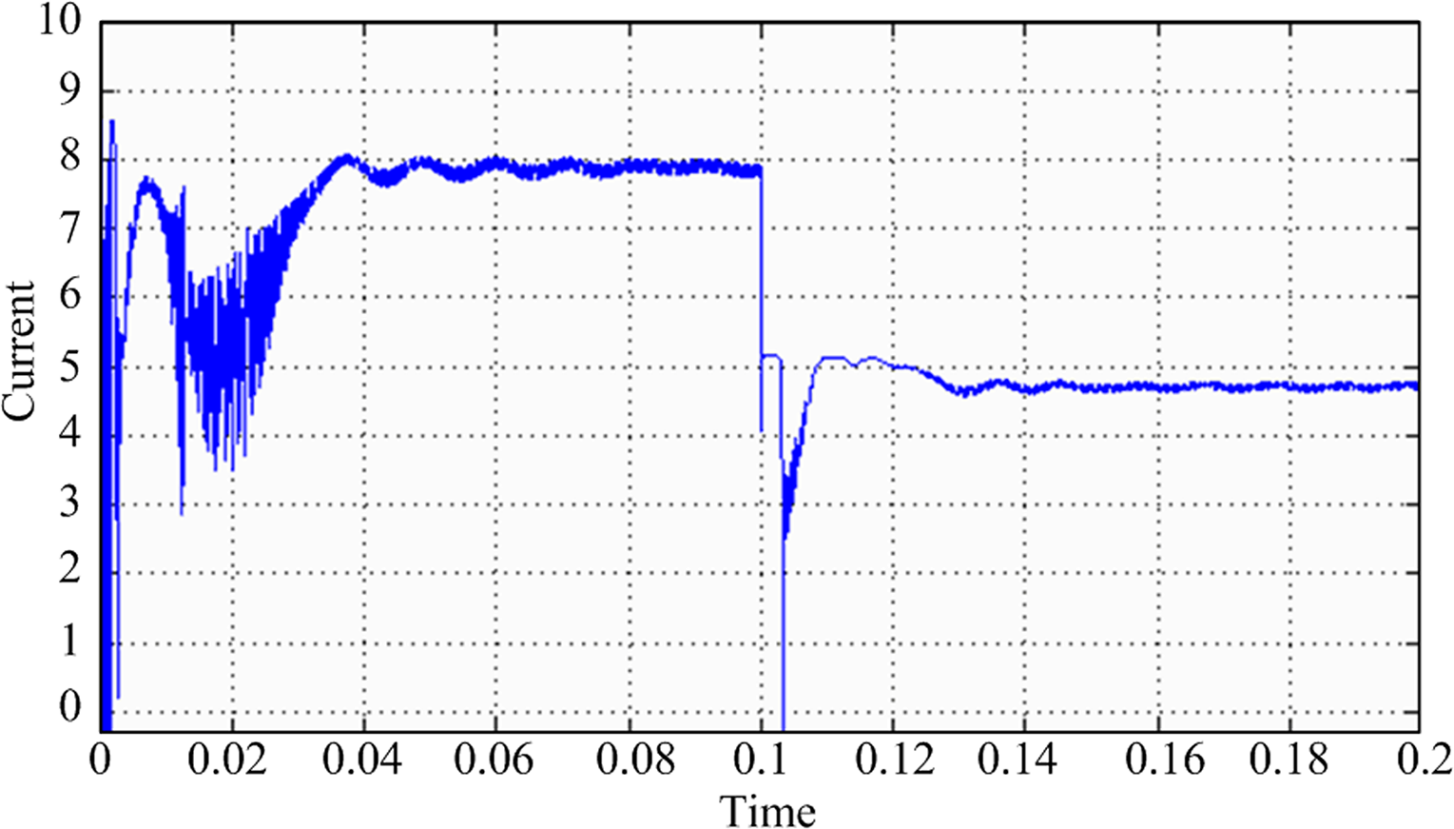
Solar cell output current *Ipv* curve.

**Fig 21:**
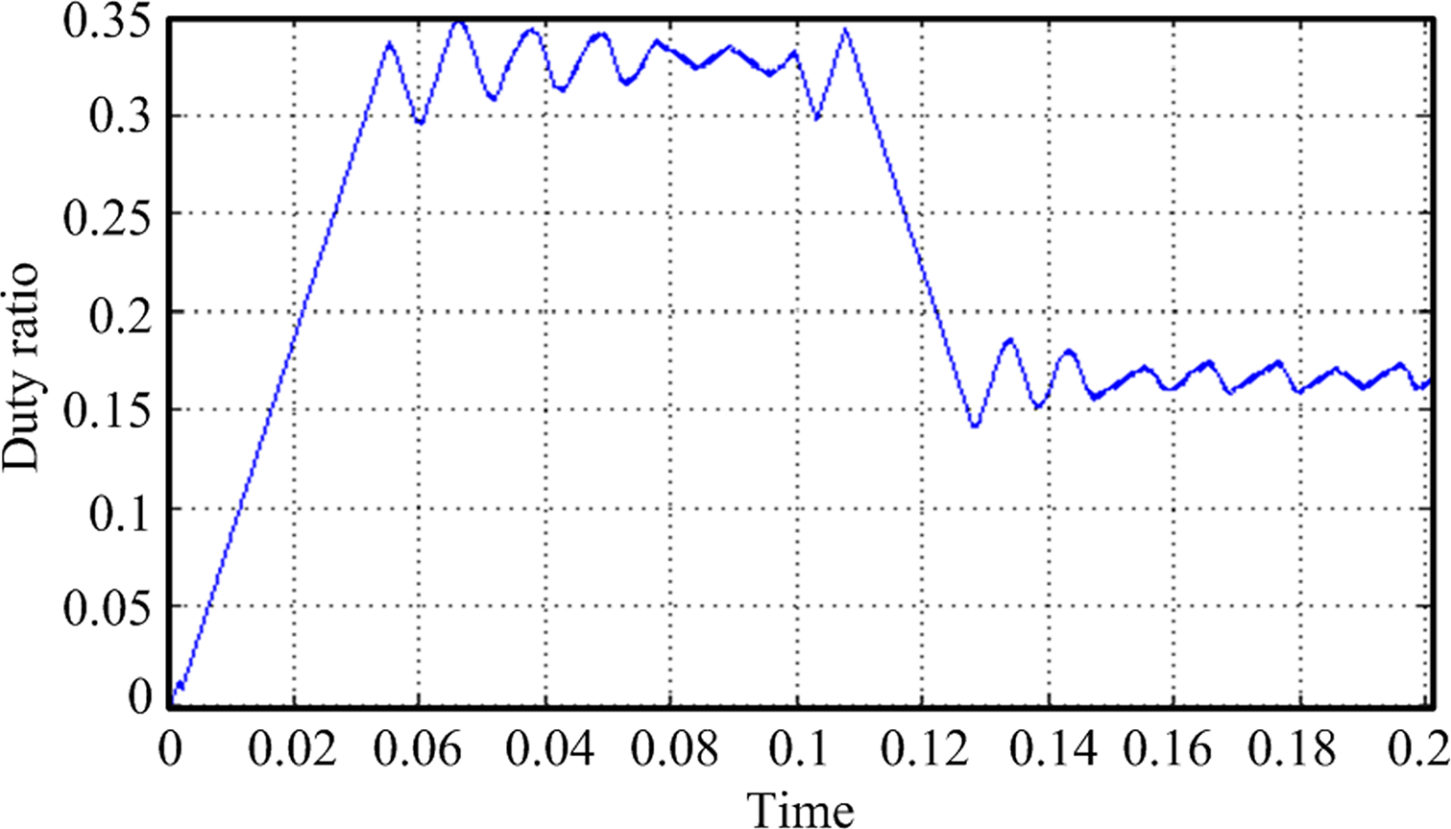
Duty-cycle curve.

**Fig 22:**
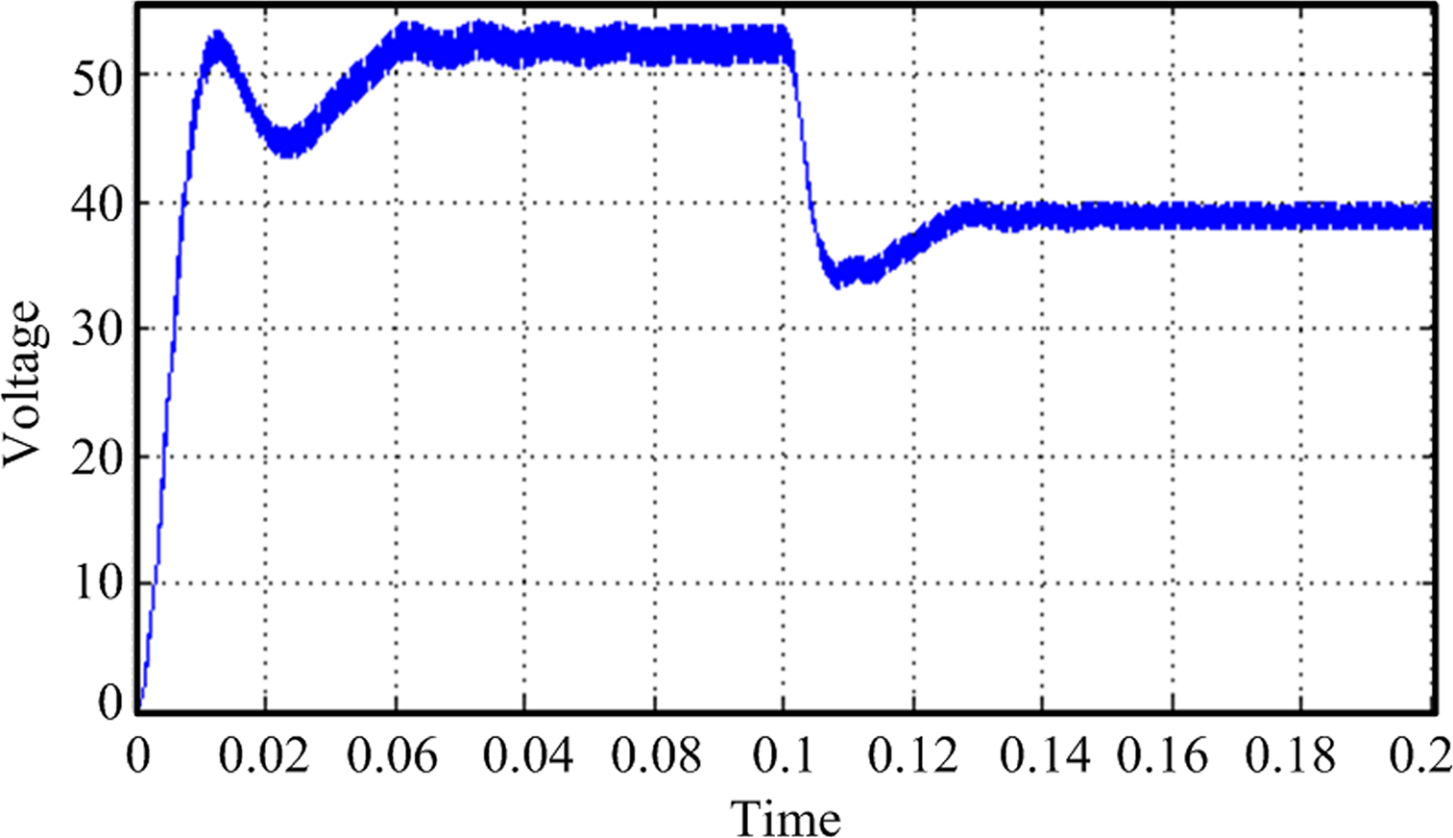
Tracking voltage curve of the maximum power point.

In this design, the MPPT mathematical simulation is modeled using the Simulink software package, which comes from the MATLAB software, according to the mathematical model of the solar cells. MPPT is performed by a fixed-step-size perturbation observation method. The initial light conditions are set to be 1 kW/m^2^, the sampling frequency is 1 kHz, and the step length is 5/1000. At the 0.1-s time point, the light intensity drops from 1 to 0.6 kW/m^2^. The *Ipv* waveform changes, as shown in Fig 20. The duty-ratio curve is shown in Fig 21. The output voltage of the maximum power modulation is shown in Fig 22, and the data of Fig 22 were Table A in S1 File.

### Improved MATLAB disturbance observation model

**Fig 23:**
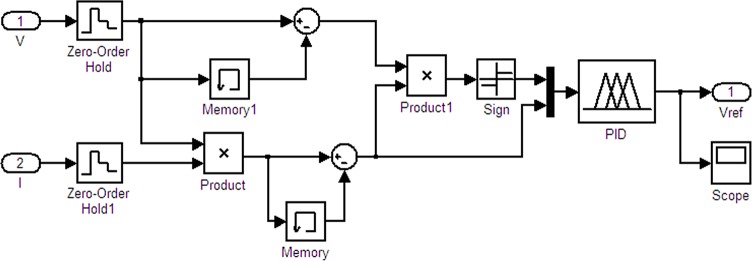
Improved disturbance observation model.

**Fig 24:**
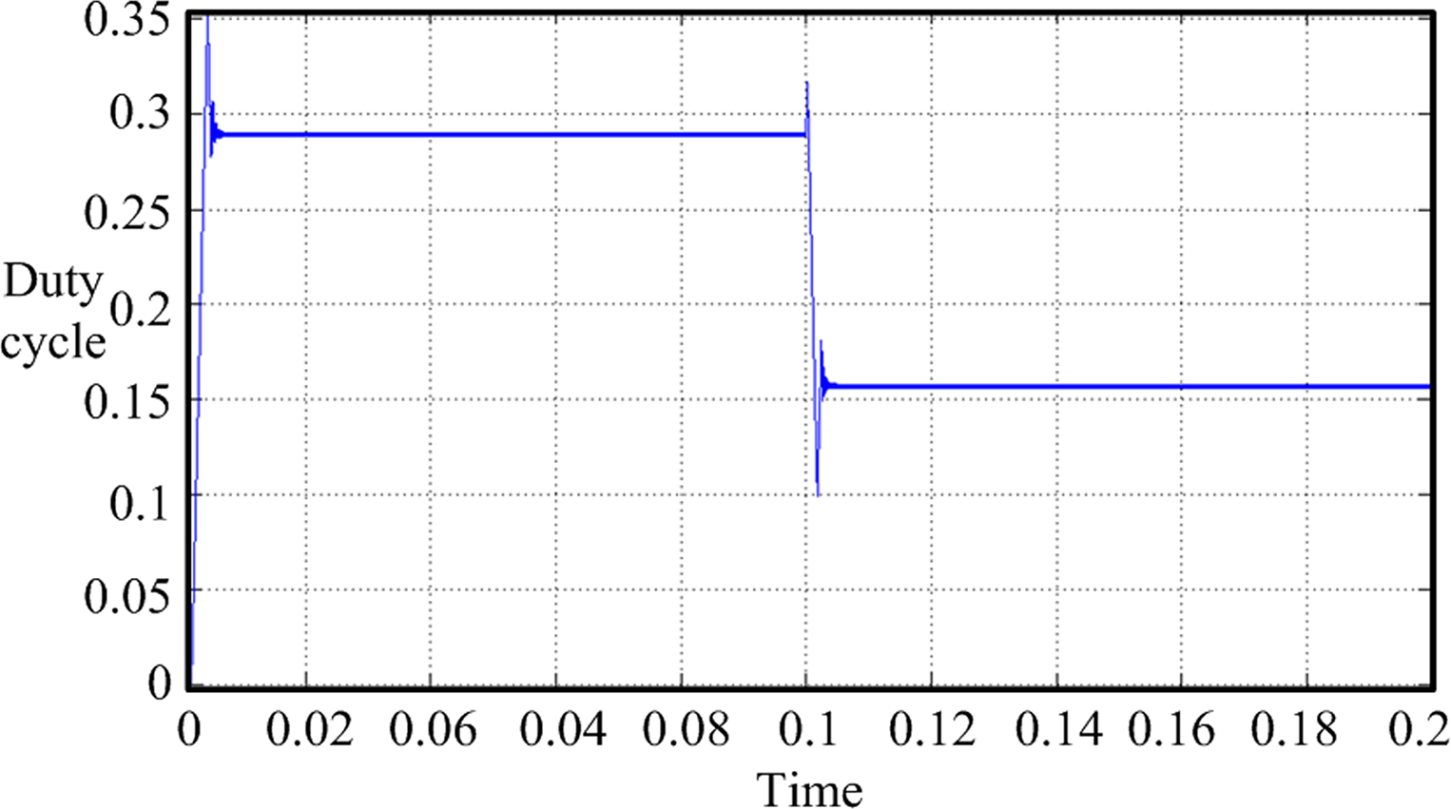
Duty-ratio curve of the improved model.

**Fig 25:**
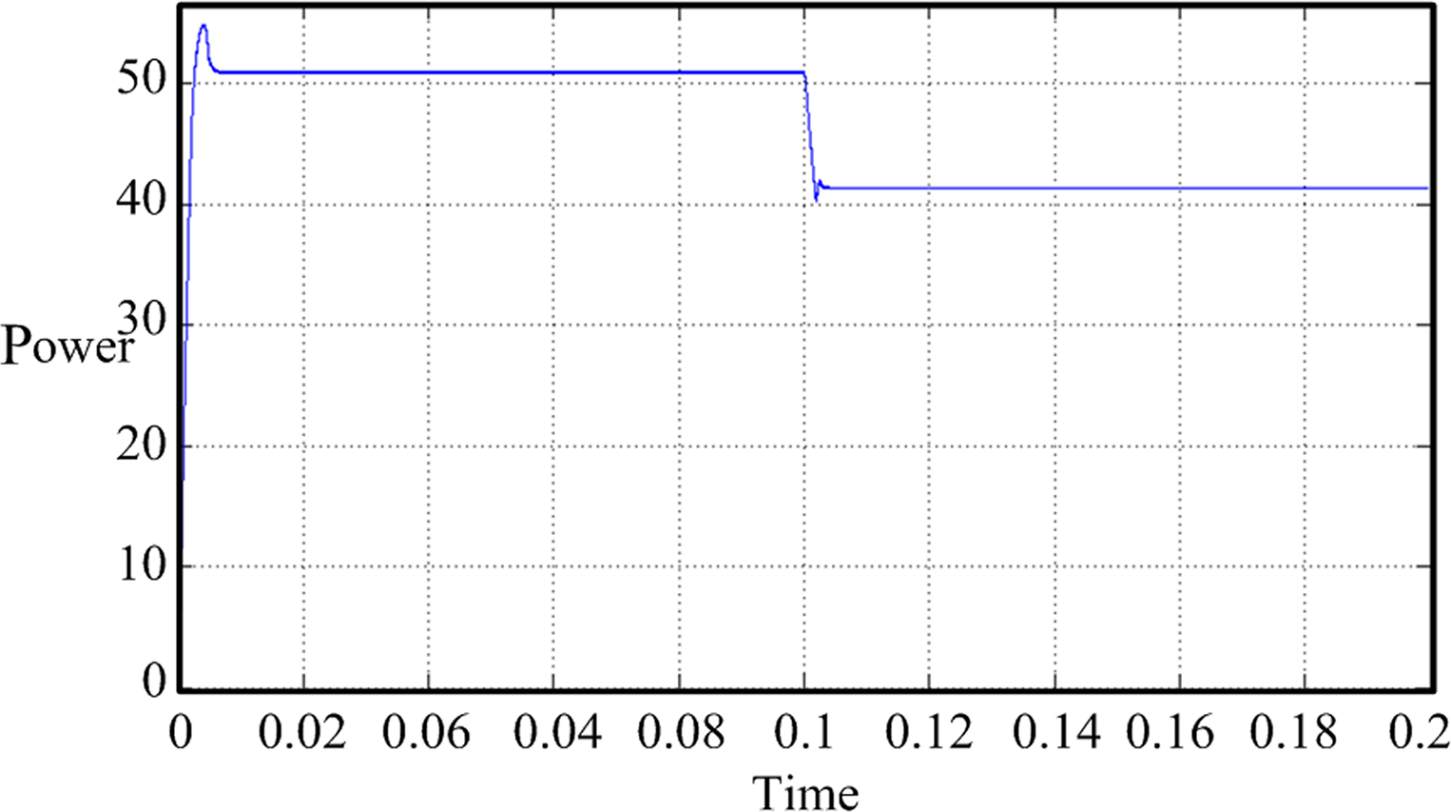
Improved MPPT voltage curve.

The simulation model that uses a fixed-step-size perturbation observation to achieve MPPT suffers from some problems, i.e., the duty-ratio adjustment process overshoots, and after achieving dynamic balance, the duty ratio and the output voltage have a larger amplitude of oscillation. To overcome these shortcomings, the disturbance observation method is improved. By combining a PID control link, the duty-ratio-adjusted step length is gradually shortened as the maximum power point changes to reduce the overshoot and oscillation amplitude. Fig 23 shows the improved disturbance observation method MPPT model. The PID control link is added to the Simulink simulation model, and its programming details are embedded in the file FUZZY_PID.fis. The duty-ratio curve of the improved model is shown in Fig 24 and the data of Fig 24 were in Table B in S1 File. The improved MPPT voltage curve is shown in Fig 25.

By comparing the improved simulation waveform with the unimproved one, we can see that after the improvement, the duty-ratio overshoot and steady-state oscillation amplitude obviously decrease. The duty-ratio value and output voltage of the improved model tend to be stable after the model reaches the steady state. The problems of vibration and overshoot are obviously reduced.

### System power-consumption test

To obtain the system power-consumption data, a measurement experiment was performed to measure the power consumption of each device module, as listed in Table 1.

**Table 1:** Average power consumption of the system device parts.

Equipment	LCD	Sensors and laser tube	Level motor and drive circuit	Vertical motor and drive circuit
**Power (mW)**	824.2	134 8.9	106 1.7	158 8.7

The system adopts a low-power design; hence, the energy consumption of each device is obtained by converting its actual operation time. The experimental result shows that the system mechanical structure level adjustment from zero to the maximum limit requires a time of 5 s, and the pitch adjustment from zero to the maximum limit requires 9 s, which means that every 10 min, the longest running time of the system is 9 s. The power consumption of each module is listed in Table 2.

**Table 2:** Average daily power consumption of the system equipment parts.

System calibration frequency (s)	Horizontal maximum correction time (s)	Pitch correction time (s)	Motor and drive circuit power consumption (mW·h)	Sensors and laser tube power consumption (mW·h)	LCD power consumption (mW·h)	Total power consumption (mW·h)
72	5	9	538.5	242.8	148.5	929.65

### Measured system charging efficiency

**Fig 26:**
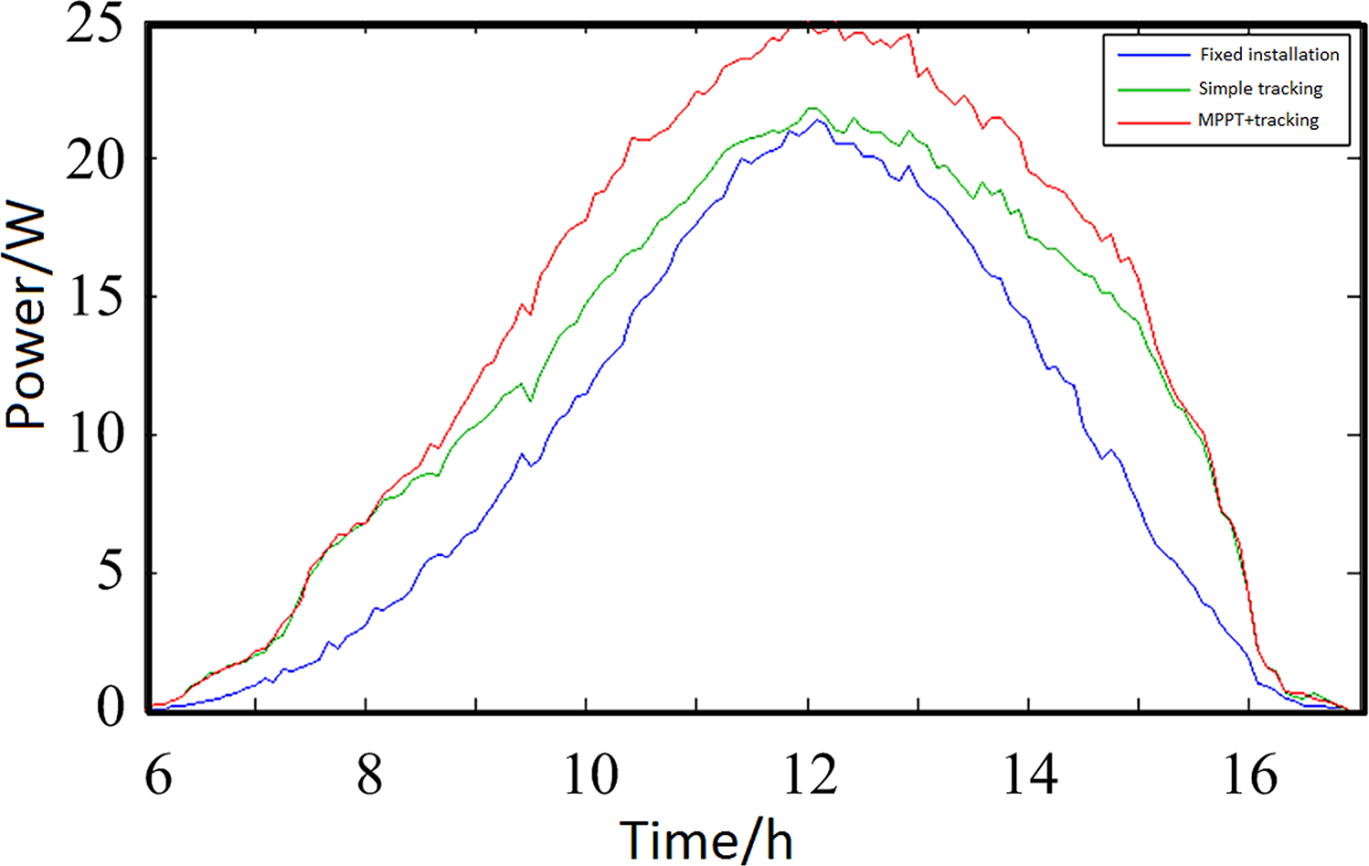
First comparison curves of the measured charging efficiency.

**Fig 27:**
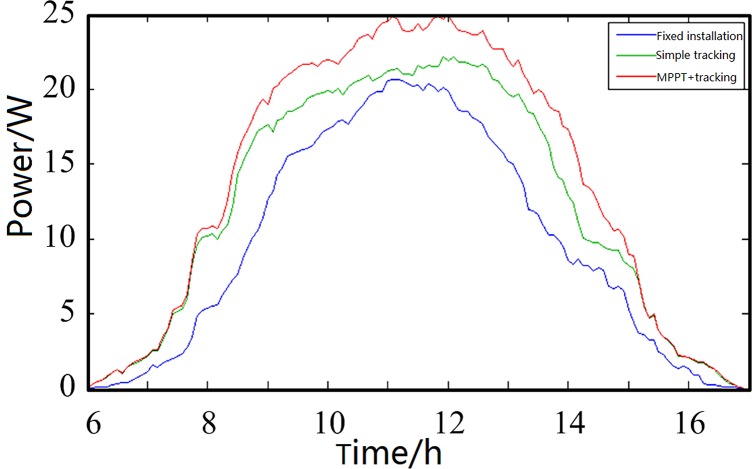
Second comparison curves of the measured charging efficiency.

**Fig 28:**
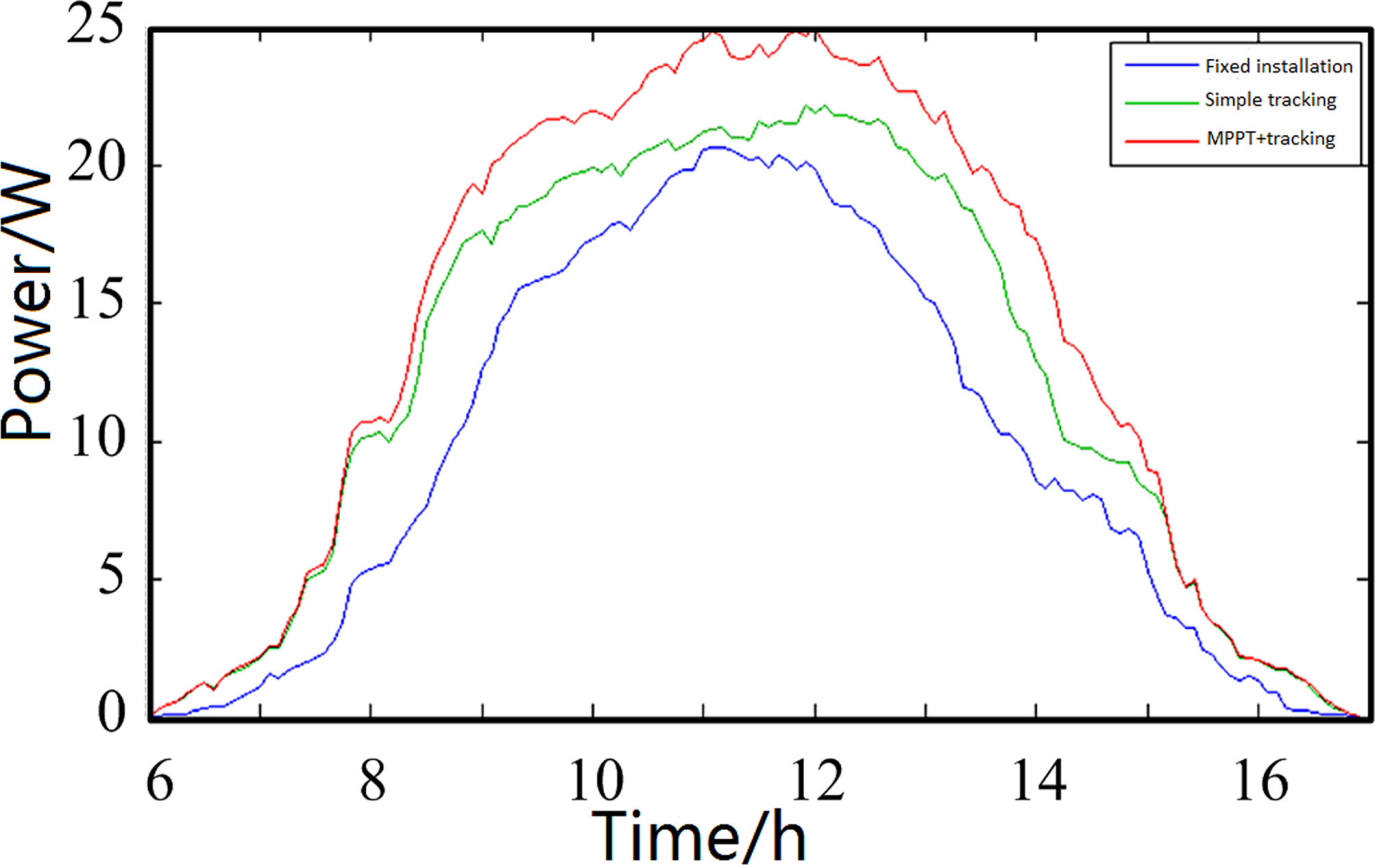
Third comparison curves of the measured charging efficiency.

The measurement experiment selected 30-W monocrystalline silicon solar panels with identical parameters. These solar panels were installed on a 45° south-facing fixed bracket with a pure tracking system and a tracking system that uses the MPPT technology. Each panel charged the same type of 12-V, 55-Ah battery, which was sufficiently discharged to the same state. When the experiment was in progress, the current entering the battery and the terminal voltage were detected in real time. The electric energy received by the battery at this moment was the net energy stored after considering the system power consumption. The current output power of the PV system and its energy storage can be obtained by calculation. The measurement experiment chose three discontinuous days, charging the system for 11 h from 06:00 to 17:00, and recorded a set of output data every 5 min for a total of 133 sets of data. A time power curve was then drawn, as shown in Fig 26, Fig27 and Fig 28, the data of Fig 26, Fig 27 and Fig 28 were in Tables C, D and E in S1 File. The system daily output can be calculated by integrating the power over time, as listed in Table 3.

**Table 3:** Comparison of the total power charging.

State	Fixed installation	Simple tracking		MPPT	
	Total amount (W·h)	Total amount (W·h)	Relative fixed installation efficiency improvement	Total amount (W·h)	Relative fixed installation efficiency improvement
**First test**	103.17	130.32	26.32%	149.07	44.49%
**Second test**	105.79	134.76	28.33%	152.25	43.92%
**Third test**	90.54	107.37	18.59%	128.16	41.55%
**Average**	99.83	124.15	24.36%	143.16	43.40%

Fig 26 shows the system charging efficiency comparison curves of the first measurement experiment from 06:00 to 17:00. The measurement conditions were as follows: temperature, from −9 to 2°C; relative humidity, 59%–79%; weather, fine.

Fig 27 shows the system charging efficiency comparison curves of the second measurement experiment from 06:00 to 17:00. The measurement conditions were as follows: temperature, from −7 to 5°C; relative humidity, 60%–90%; weather, fine.

Fig 28 shows the system charging efficiency comparison curves of the third measurement experiment from 06:00 to 17:00. The measurement conditions were as follows: temperature, from −6 to 6°C; relative humidity, 60%–85%; weather, clear to overcast. Fig 28 shows that the weather markedly changed at approximately 08:30; the output curve when the sun was covered by clouds is circled in the figure.

The results of the three measurement experiments tended to be the same. This design of the PV system yielded the highest efficiency and could exhibit higher power generation efficiency improvement under different weather conditions. The system efficiency increase could reach 44.49% compared with that of the panels with fixed installation. The efficiency could still increase by 41.55% in a relatively bad rainy day, which proves that the influence of outside conditions is relatively small, and this system is stable and reliable.

## Conclusion

This paper has presented the study and summary of the utilization and prospects of solar PV cells at home and abroad and has pointed out the significance of the PV power generation technology research. In addition, this paper has presented the profound study of the efficiency of the solar tracking and MPPT technology to enhance the mechanism. We designed the system capacity and provided a system technology target.

Related experiments and simulations to test the system performance as well as the parameters were designed. A Simulink simulation model of the disturbance observation method was designed. By comparing the performance of the model before and after the improvement, the superiority of the improved algorithm was proven. A performance-testing experiment on the sun-positioning recognition module was designed. This experiment tested the tracking accuracy and perspective of the module, the experimental verified that the photoelectric sensor resolution can reach 0.344° and the maximum tracking error was less than 2.5°. Finally, a charge–discharge and power-consumption testing experiment was designed, which proved the rationality of the system, the stability of operation, and the high generating efficiency,the experimental verified that the power consumption of system perihterals was under 1W·h a day, and the largest improvement in the charge efficiency could reach 44.5%.

## Supporting Information

S1 File Dataset. (DOC)
