# Supplementary material for: Intelligent Photovoltaic Systems by Combining the Improved Perturbation Method of Observation and Sun Location Tracking
Source: PLoS One. 2016 Jun 21;11(6):e0156858. doi: 10.1371/journal.pone.0156858 (PMC4915704; doi:10.1371/journal.pone.0156858)
Supplement: S1 File — (DOC) [file pone.0156858.s001.doc]

**S1 File**

**Data set**

**Table A**

| 2.00E-03 | 9.24E+00 |
| --- | --- |
| 4.00E-03 | 2.24E+01 |
| 6.01E-03 | 3.37E+01 |
| 8.01E-03 | 4.42E+01 |
| 1.00E-02 | 5.07E+01 |
| 1.20E-02 | 5.21E+01 |
| 1.40E-02 | 5.12E+01 |
| 1.60E-02 | 4.90E+01 |
| 1.80E-02 | 4.78E+01 |
| 1.80E-02 | 4.67E+01 |
| 2.00E-02 | 4.53E+01 |
| 2.20E-02 | 4.47E+01 |
| 2.40E-02 | 4.49E+01 |
| 2.60E-02 | 4.55E+01 |
| 2.80E-02 | 4.63E+01 |
| 3.00E-02 | 4.75E+01 |
| 3.20E-02 | 4.84E+01 |
| 3.40E-02 | 4.94E+01 |
| 3.60E-02 | 5.03E+01 |
| 3.80E-02 | 5.16E+01 |
| 4.00E-02 | 5.23E+01 |
| 4.20E-02 | 5.22E+01 |
| 4.40E-02 | 5.21E+01 |
| 4.60E-02 | 5.18E+01 |
| 4.80E-02 | 5.18E+01 |
| 5.00E-02 | 5.22E+01 |
| 5.20E-02 | 5.30E+01 |
| 5.40E-02 | 5.25E+01 |
| 5.60E-02 | 5.22E+01 |
| 5.80E-02 | 5.18E+01 |
| 6.00E-02 | 5.21E+01 |
| 6.20E-02 | 5.23E+01 |
| 6.40E-02 | 5.25E+01 |
| 6.60E-02 | 5.24E+01 |
| 6.80E-02 | 5.20E+01 |
| 7.00E-02 | 5.18E+01 |
| 7.20E-02 | 5.22E+01 |
| 7.40E-02 | 5.25E+01 |
| 7.60E-02 | 5.23E+01 |
| 7.80E-02 | 5.23E+01 |
| 8.00E-02 | 5.22E+01 |
| 8.20E-02 | 5.24E+01 |
| 8.40E-02 | 5.21E+01 |
| 8.60E-02 | 5.23E+01 |
| 8.80E-02 | 5.23E+01 |
| 9.00E-02 | 5.21E+01 |
| 9.20E-02 | 5.26E+01 |
| 9.40E-02 | 5.22E+01 |
| 9.60E-02 | 5.23E+01 |
| 9.80E-02 | 5.19E+01 |
| 1.00E-01 | 5.18E+01 |
| 1.02E-01 | 4.71E+01 |
| 1.04E-01 | 3.89E+01 |
| 1.06E-01 | 3.51E+01 |
| 1.08E-01 | 3.45E+01 |
| 1.10E-01 | 3.46E+01 |
| 1.12E-01 | 3.45E+01 |
| 1.14E-01 | 3.49E+01 |
| 1.16E-01 | 3.58E+01 |
| 1.18E-01 | 3.65E+01 |
| 1.20E-01 | 3.68E+01 |
| 1.22E-01 | 3.75E+01 |
| 1.24E-01 | 3.80E+01 |
| 1.26E-01 | 3.87E+01 |
| 1.28E-01 | 3.90E+01 |
| 1.30E-01 | 3.91E+01 |
| 1.32E-01 | 3.90E+01 |
| 1.34E-01 | 3.89E+01 |
| 1.36E-01 | 3.87E+01 |
| 1.38E-01 | 3.88E+01 |
| 1.40E-01 | 3.90E+01 |
| 1.42E-01 | 3.88E+01 |
| 1.44E-01 | 3.89E+01 |
| 1.46E-01 | 3.90E+01 |
| 1.48E-01 | 3.91E+01 |
| 1.50E-01 | 3.88E+01 |
| 1.52E-01 | 3.87E+01 |
| 1.54E-01 | 3.88E+01 |
| 1.56E-01 | 3.88E+01 |
| 1.58E-01 | 3.91E+01 |
| 1.60E-01 | 3.91E+01 |
| 1.62E-01 | 3.89E+01 |
| 1.64E-01 | 3.87E+01 |
| 1.66E-01 | 3.88E+01 |
| 1.68E-01 | 3.89E+01 |
| 1.70E-01 | 3.91E+01 |
| 1.72E-01 | 3.91E+01 |
| 1.74E-01 | 3.90E+01 |
| 1.76E-01 | 3.88E+01 |
| 1.78E-01 | 3.87E+01 |
| 1.80E-01 | 3.89E+01 |
| 1.82E-01 | 3.89E+01 |
| 1.84E-01 | 3.90E+01 |
| 1.86E-01 | 3.89E+01 |
| 1.88E-01 | 3.90E+01 |
| 1.90E-01 | 3.87E+01 |
| 1.92E-01 | 3.87E+01 |
| 1.94E-01 | 3.88E+01 |
| 1.96E-01 | 3.88E+01 |
| 1.98E-01 | 3.91E+01 |

**Table B**

| 5.17E-04 | 1.35E-02 |
| --- | --- |
| 5.17E-04 | 1.60E-02 |
| 5.17E-04 | 1.85E-02 |
| 5.17E-04 | 2.10E-02 |
| 5.17E-04 | 2.36E-02 |
| 5.17E-04 | 2.61E-02 |
| 5.17E-04 | 2.86E-02 |
| 5.17E-04 | 3.11E-02 |
| 5.17E-04 | 3.37E-02 |
| 5.17E-04 | 3.62E-02 |
| 4.89E-04 | 3.87E-02 |
| 4.89E-04 | 4.12E-02 |
| 5.75E-04 | 4.37E-02 |
| 6.90E-04 | 4.61E-02 |
| 7.76E-04 | 4.86E-02 |
| 8.05E-04 | 5.11E-02 |
| 7.76E-04 | 5.37E-02 |
| 7.76E-04 | 5.62E-02 |
| 7.76E-04 | 5.87E-02 |
| 7.76E-04 | 6.12E-02 |
| 7.76E-04 | 6.38E-02 |
| 7.76E-04 | 6.63E-02 |
| 7.76E-04 | 6.88E-02 |
| 7.47E-04 | 7.13E-02 |
| 7.47E-04 | 7.39E-02 |
| 8.34E-04 | 7.63E-02 |
| 9.49E-04 | 7.87E-02 |
| 1.03E-03 | 8.12E-02 |
| 1.03E-03 | 8.38E-02 |
| 1.03E-03 | 8.63E-02 |
| 1.03E-03 | 8.88E-02 |
| 1.03E-03 | 9.13E-02 |
| 1.03E-03 | 9.39E-02 |
| 1.03E-03 | 9.64E-02 |
| 1.03E-03 | 9.89E-02 |
| 1.03E-03 | 1.01E-01 |
| 1.03E-03 | 1.04E-01 |
| 1.03E-03 | 1.06E-01 |
| 5.17E-04 | 1.09E-02 |
| 5.17E-04 | 8.41E-03 |
| 5.17E-04 | 5.89E-03 |
| 5.17E-04 | 3.37E-03 |
| 1.03E-03 | 1.09E-01 |
| 1.29E-03 | 1.11E-01 |
| 1.32E-03 | 1.13E-01 |
| 1.29E-03 | 1.16E-01 |
| 1.29E-03 | 1.18E-01 |
| 1.29E-03 | 1.21E-01 |
| 1.29E-03 | 1.23E-01 |
| 1.29E-03 | 1.26E-01 |
| 1.29E-03 | 1.29E-01 |
| 1.29E-03 | 1.31E-01 |
| 1.29E-03 | 1.34E-01 |
| 1.26E-03 | 1.36E-01 |
| 1.26E-03 | 1.39E-01 |
| 1.35E-03 | 1.41E-01 |
| 1.47E-03 | 1.43E-01 |
| 1.55E-03 | 1.46E-01 |
| 1.55E-03 | 1.49E-01 |
| 1.55E-03 | 1.51E-01 |
| 1.55E-03 | 1.54E-01 |
| 1.55E-03 | 1.56E-01 |
| 1.55E-03 | 1.59E-01 |
| 1.55E-03 | 1.61E-01 |
| 1.55E-03 | 1.64E-01 |
| 1.55E-03 | 1.66E-01 |
| 1.55E-03 | 1.69E-01 |
| 1.55E-03 | 1.71E-01 |
| 1.03E-03 | 1.06E-01 |
| 1.81E-03 | 1.75E-01 |
| 1.81E-03 | 1.78E-01 |
| 1.81E-03 | 1.80E-01 |
| 1.81E-03 | 1.83E-01 |
| 1.81E-03 | 1.85E-01 |
| 1.81E-03 | 1.88E-01 |
| 1.81E-03 | 1.90E-01 |
| 1.81E-03 | 1.93E-01 |
| 1.81E-03 | 1.95E-01 |
| 1.81E-03 | 1.98E-01 |
| 1.78E-03 | 2.00E-01 |
| 1.78E-03 | 2.03E-01 |
| 1.87E-03 | 2.05E-01 |
| 1.98E-03 | 2.08E-01 |
| 2.07E-03 | 2.10E-01 |
| 2.07E-03 | 2.13E-01 |
| 2.07E-03 | 2.15E-01 |
| 2.07E-03 | 2.18E-01 |
| 2.07E-03 | 2.20E-01 |
| 2.07E-03 | 2.23E-01 |
| 2.07E-03 | 2.25E-01 |
| 2.07E-03 | 2.28E-01 |
| 2.07E-03 | 2.30E-01 |
| 2.04E-03 | 2.33E-01 |
| 2.04E-03 | 2.35E-01 |
| 2.13E-03 | 2.38E-01 |
| 2.24E-03 | 2.40E-01 |
| 2.33E-03 | 2.43E-01 |
| 2.33E-03 | 2.45E-01 |
| 2.30E-03 | 2.48E-01 |
| 2.30E-03 | 2.50E-01 |
| 2.33E-03 | 2.53E-01 |
| 2.33E-03 | 2.55E-01 |
| 2.33E-03 | 2.58E-01 |
| 2.33E-03 | 2.60E-01 |
| 2.33E-03 | 2.63E-01 |
| 2.30E-03 | 2.65E-01 |
| 2.30E-03 | 2.68E-01 |
| 2.39E-03 | 2.70E-01 |
| 2.50E-03 | 2.73E-01 |
| 2.59E-03 | 2.75E-01 |
| 2.59E-03 | 2.78E-01 |
| 2.59E-03 | 2.80E-01 |
| 2.59E-03 | 2.83E-01 |
| 2.59E-03 | 2.85E-01 |
| 2.59E-03 | 2.88E-01 |
| 2.59E-03 | 2.91E-01 |
| 2.59E-03 | 2.93E-01 |
| 2.59E-03 | 2.96E-01 |
| 2.56E-03 | 2.98E-01 |
| 2.56E-03 | 3.01E-01 |
| 2.64E-03 | 3.03E-01 |
| 2.76E-03 | 3.06E-01 |
| 2.85E-03 | 3.08E-01 |
| 2.85E-03 | 3.11E-01 |
| 2.85E-03 | 3.13E-01 |
| 2.85E-03 | 3.16E-01 |
| 2.85E-03 | 3.18E-01 |
| 2.85E-03 | 3.21E-01 |
| 2.85E-03 | 3.23E-01 |
| 2.85E-03 | 3.26E-01 |
| 2.85E-03 | 3.28E-01 |
| 2.87E-03 | 3.31E-01 |
| 2.96E-03 | 3.33E-01 |
| 3.08E-03 | 3.36E-01 |
| 3.13E-03 | 3.38E-01 |
| 3.10E-03 | 3.41E-01 |
| 3.10E-03 | 3.43E-01 |
| 3.10E-03 | 3.46E-01 |
| 3.10E-03 | 3.48E-01 |
| 3.08E-03 | 3.51E-01 |
| 1.55E-03 | 1.73E-01 |
| 1.55E-03 | 1.70E-01 |
| 3.36E-03 | 3.33E-01 |
| 2.85E-03 | 3.31E-01 |
| 3.36E-03 | 3.29E-01 |
| 3.36E-03 | 3.31E-01 |
| 3.36E-03 | 3.34E-01 |
| 3.36E-03 | 3.26E-01 |
| 3.36E-03 | 3.24E-01 |
| 3.36E-03 | 3.21E-01 |
| 3.36E-03 | 3.19E-01 |
| 3.36E-03 | 3.16E-01 |
| 3.33E-03 | 3.14E-01 |
| 3.36E-03 | 3.11E-01 |
| 3.45E-03 | 3.09E-01 |
| 3.59E-03 | 3.07E-01 |
| 3.68E-03 | 3.04E-01 |
| 3.68E-03 | 3.01E-01 |
| 3.74E-03 | 2.99E-01 |
| 3.91E-03 | 2.96E-01 |
| 4.02E-03 | 2.94E-01 |
| 3.74E-03 | 2.92E-01 |
| 2.82E-03 | 3.27E-01 |
| 3.62E-03 | 2.79E-01 |
| 3.62E-03 | 2.82E-01 |
| 3.71E-03 | 2.84E-01 |
| 3.94E-03 | 2.87E-01 |
| 4.25E-03 | 2.89E-01 |
| 4.57E-03 | 2.91E-01 |
| 5.43E-03 | 2.89E-01 |
| 6.21E-03 | 2.89E-01 |
| 6.99E-03 | 2.89E-01 |
| 7.76E-03 | 2.89E-01 |
| 8.54E-03 | 2.89E-01 |
| 9.31E-03 | 2.89E-01 |
| 1.01E-02 | 2.89E-01 |
| 1.09E-02 | 2.89E-01 |
| 1.16E-02 | 2.89E-01 |
| 1.24E-02 | 2.89E-01 |
| 1.32E-02 | 2.89E-01 |
| 1.40E-02 | 2.89E-01 |
| 1.47E-02 | 2.89E-01 |
| 1.55E-02 | 2.89E-01 |
| 1.63E-02 | 2.89E-01 |
| 1.71E-02 | 2.89E-01 |
| 1.79E-02 | 2.89E-01 |
| 1.86E-02 | 2.89E-01 |
| 1.94E-02 | 2.89E-01 |
| 2.02E-02 | 2.89E-01 |
| 2.10E-02 | 2.89E-01 |
| 2.17E-02 | 2.89E-01 |
| 2.25E-02 | 2.89E-01 |
| 2.33E-02 | 2.89E-01 |
| 2.41E-02 | 2.89E-01 |
| 2.48E-02 | 2.89E-01 |
| 2.56E-02 | 2.89E-01 |
| 2.64E-02 | 2.89E-01 |
| 2.72E-02 | 2.89E-01 |
| 2.79E-02 | 2.89E-01 |
| 2.87E-02 | 2.89E-01 |
| 2.95E-02 | 2.89E-01 |
| 3.03E-02 | 2.89E-01 |
| 3.10E-02 | 2.89E-01 |
| 3.18E-02 | 2.89E-01 |
| 3.26E-02 | 2.89E-01 |
| 3.34E-02 | 2.89E-01 |
| 3.42E-02 | 2.89E-01 |
| 3.49E-02 | 2.89E-01 |
| 3.57E-02 | 2.89E-01 |
| 3.65E-02 | 2.89E-01 |
| 3.73E-02 | 2.89E-01 |
| 3.80E-02 | 2.89E-01 |
| 3.88E-02 | 2.89E-01 |
| 3.96E-02 | 2.89E-01 |
| 4.04E-02 | 2.89E-01 |
| 4.11E-02 | 2.89E-01 |
| 4.19E-02 | 2.89E-01 |
| 4.27E-02 | 2.89E-01 |
| 4.35E-02 | 2.89E-01 |
| 4.42E-02 | 2.89E-01 |
| 4.50E-02 | 2.89E-01 |
| 4.58E-02 | 2.89E-01 |
| 4.66E-02 | 2.89E-01 |
| 4.73E-02 | 2.89E-01 |
| 4.81E-02 | 2.89E-01 |
| 4.89E-02 | 2.89E-01 |
| 4.97E-02 | 2.89E-01 |
| 5.05E-02 | 2.89E-01 |
| 5.12E-02 | 2.89E-01 |
| 5.20E-02 | 2.89E-01 |
| 5.28E-02 | 2.89E-01 |
| 5.36E-02 | 2.89E-01 |
| 5.43E-02 | 2.89E-01 |
| 5.51E-02 | 2.89E-01 |
| 5.59E-02 | 2.89E-01 |
| 5.67E-02 | 2.89E-01 |
| 5.74E-02 | 2.89E-01 |
| 5.82E-02 | 2.89E-01 |
| 5.90E-02 | 2.89E-01 |
| 5.98E-02 | 2.89E-01 |
| 6.05E-02 | 2.89E-01 |
| 6.13E-02 | 2.89E-01 |
| 6.21E-02 | 2.89E-01 |
| 6.29E-02 | 2.89E-01 |
| 6.36E-02 | 2.89E-01 |
| 6.44E-02 | 2.89E-01 |
| 6.52E-02 | 2.89E-01 |
| 6.60E-02 | 2.89E-01 |
| 6.68E-02 | 2.89E-01 |
| 6.75E-02 | 2.89E-01 |
| 6.83E-02 | 2.89E-01 |
| 6.91E-02 | 2.89E-01 |
| 6.99E-02 | 2.89E-01 |
| 7.06E-02 | 2.89E-01 |
| 7.14E-02 | 2.89E-01 |
| 7.22E-02 | 2.89E-01 |
| 7.30E-02 | 2.89E-01 |
| 7.37E-02 | 2.89E-01 |
| 7.45E-02 | 2.89E-01 |
| 7.53E-02 | 2.89E-01 |
| 7.61E-02 | 2.89E-01 |
| 7.68E-02 | 2.89E-01 |
| 7.76E-02 | 2.89E-01 |
| 7.84E-02 | 2.89E-01 |
| 7.92E-02 | 2.89E-01 |
| 7.99E-02 | 2.89E-01 |
| 8.07E-02 | 2.89E-01 |
| 8.15E-02 | 2.89E-01 |
| 8.23E-02 | 2.89E-01 |
| 8.31E-02 | 2.89E-01 |
| 8.38E-02 | 2.89E-01 |
| 8.46E-02 | 2.89E-01 |
| 8.54E-02 | 2.89E-01 |
| 8.62E-02 | 2.89E-01 |
| 8.69E-02 | 2.89E-01 |
| 8.77E-02 | 2.89E-01 |
| 8.85E-02 | 2.89E-01 |
| 8.93E-02 | 2.89E-01 |
| 9.00E-02 | 2.89E-01 |
| 9.08E-02 | 2.89E-01 |
| 9.16E-02 | 2.89E-01 |
| 9.24E-02 | 2.89E-01 |
| 9.31E-02 | 2.89E-01 |
| 9.39E-02 | 2.89E-01 |
| 9.47E-02 | 2.89E-01 |
| 9.55E-02 | 2.89E-01 |
| 9.62E-02 | 2.89E-01 |
| 9.70E-02 | 2.89E-01 |
| 9.78E-02 | 2.89E-01 |
| 9.86E-02 | 2.90E-01 |
| 9.92E-02 | 2.91E-01 |
| 9.95E-02 | 2.93E-01 |
| 9.96E-02 | 2.95E-01 |
| 9.95E-02 | 2.98E-01 |
| 9.94E-02 | 3.00E-01 |
| 9.94E-02 | 3.03E-01 |
| 9.93E-02 | 3.05E-01 |
| 9.93E-02 | 3.08E-01 |
| 9.92E-02 | 3.10E-01 |
| 9.92E-02 | 3.13E-01 |
| 9.94E-02 | 3.15E-01 |
| 4.66E-03 | 2.89E-01 |
| 3.91E-03 | 2.90E-01 |
| 9.96E-02 | 2.89E-01 |
| 9.96E-02 | 2.92E-01 |
| 9.96E-02 | 2.87E-01 |
| 9.96E-02 | 2.84E-01 |
| 9.96E-02 | 2.82E-01 |
| 9.96E-02 | 2.79E-01 |
| 9.96E-02 | 2.77E-01 |
| 9.96E-02 | 2.74E-01 |
| 9.96E-02 | 2.72E-01 |
| 9.96E-02 | 2.69E-01 |
| 9.97E-02 | 2.67E-01 |
| 9.97E-02 | 2.64E-01 |
| 9.97E-02 | 2.62E-01 |
| 9.97E-02 | 2.59E-01 |
| 9.98E-02 | 2.57E-01 |
| 9.98E-02 | 2.54E-01 |
| 9.98E-02 | 2.52E-01 |
| 9.98E-02 | 2.49E-01 |
| 9.97E-02 | 2.47E-01 |
| 9.97E-02 | 2.44E-01 |
| 9.97E-02 | 2.42E-01 |
| 9.98E-02 | 2.39E-01 |
| 9.99E-02 | 2.37E-01 |
| 9.99E-02 | 2.34E-01 |
| 9.99E-02 | 2.32E-01 |
| 9.99E-02 | 2.29E-01 |
| 9.99E-02 | 2.27E-01 |
| 9.99E-02 | 2.24E-01 |
| 9.99E-02 | 2.21E-01 |
| 9.99E-02 | 2.19E-01 |
| 9.99E-02 | 2.16E-01 |
| 9.99E-02 | 2.14E-01 |
| 9.99E-02 | 2.11E-01 |
| 9.99E-02 | 2.09E-01 |
| 1.00E-01 | 2.06E-01 |
| 1.00E-01 | 2.04E-01 |
| 1.00E-01 | 2.01E-01 |
| 1.00E-01 | 1.99E-01 |
| 1.00E-01 | 1.96E-01 |
| 1.00E-01 | 1.94E-01 |
| 1.00E-01 | 1.91E-01 |
| 1.00E-01 | 1.89E-01 |
| 1.00E-01 | 1.86E-01 |
| 1.00E-01 | 1.84E-01 |
| 1.00E-01 | 1.81E-01 |
| 1.00E-01 | 1.79E-01 |
| 1.00E-01 | 1.76E-01 |
| 1.00E-01 | 1.74E-01 |
| 1.00E-01 | 1.71E-01 |
| 1.00E-01 | 1.69E-01 |
| 1.00E-01 | 1.66E-01 |
| 1.00E-01 | 1.64E-01 |
| 1.00E-01 | 1.61E-01 |
| 1.00E-01 | 1.59E-01 |
| 1.00E-01 | 1.56E-01 |
| 1.00E-01 | 1.54E-01 |
| 1.00E-01 | 1.51E-01 |
| 1.00E-01 | 1.49E-01 |
| 1.00E-01 | 1.46E-01 |
| 1.00E-01 | 1.43E-01 |
| 1.00E-01 | 1.41E-01 |
| 1.01E-01 | 1.39E-01 |
| 1.01E-01 | 1.36E-01 |
| 1.01E-01 | 1.34E-01 |
| 1.01E-01 | 1.31E-01 |
| 1.01E-01 | 1.29E-01 |
| 1.01E-01 | 1.26E-01 |
| 1.01E-01 | 1.24E-01 |
| 1.01E-01 | 1.21E-01 |
| 1.01E-01 | 1.19E-01 |
| 1.01E-01 | 1.16E-01 |
| 1.01E-01 | 1.13E-01 |
| 1.01E-01 | 1.11E-01 |
| 1.01E-01 | 1.08E-01 |
| 1.01E-01 | 1.06E-01 |
| 1.01E-01 | 1.03E-01 |
| 1.01E-01 | 1.01E-01 |
| 9.91E-02 | 2.91E-01 |
| 9.88E-02 | 2.89E-01 |
| 1.01E-01 | 1.26E-01 |
| 1.01E-01 | 1.29E-01 |
| 1.01E-01 | 1.31E-01 |
| 1.01E-01 | 1.34E-01 |
| 1.01E-01 | 1.36E-01 |
| 1.01E-01 | 1.39E-01 |
| 1.01E-01 | 1.41E-01 |
| 1.01E-01 | 1.44E-01 |
| 1.01E-01 | 1.46E-01 |
| 1.01E-01 | 1.49E-01 |
| 1.01E-01 | 1.51E-01 |
| 1.01E-01 | 1.54E-01 |
| 1.01E-01 | 1.56E-01 |
| 1.01E-01 | 1.28E-01 |
| 1.01E-01 | 1.58E-01 |
| 1.02E-01 | 1.59E-01 |
| 1.03E-01 | 1.58E-01 |
| 1.04E-01 | 1.58E-01 |
| 1.04E-01 | 1.58E-01 |
| 1.05E-01 | 1.58E-01 |
| 1.06E-01 | 1.58E-01 |
| 1.07E-01 | 1.58E-01 |
| 1.08E-01 | 1.58E-01 |
| 1.08E-01 | 1.58E-01 |
| 1.09E-01 | 1.58E-01 |
| 1.10E-01 | 1.58E-01 |
| 1.11E-01 | 1.58E-01 |
| 1.11E-01 | 1.58E-01 |
| 1.12E-01 | 1.58E-01 |
| 1.13E-01 | 1.58E-01 |
| 1.14E-01 | 1.58E-01 |
| 1.15E-01 | 1.58E-01 |
| 1.15E-01 | 1.58E-01 |
| 1.16E-01 | 1.58E-01 |
| 1.17E-01 | 1.58E-01 |
| 1.18E-01 | 1.58E-01 |
| 1.18E-01 | 1.58E-01 |
| 1.19E-01 | 1.58E-01 |
| 1.20E-01 | 1.58E-01 |
| 1.21E-01 | 1.58E-01 |
| 1.22E-01 | 1.58E-01 |
| 1.22E-01 | 1.58E-01 |
| 1.23E-01 | 1.58E-01 |
| 1.24E-01 | 1.58E-01 |
| 1.25E-01 | 1.58E-01 |
| 1.25E-01 | 1.58E-01 |
| 1.26E-01 | 1.58E-01 |
| 1.27E-01 | 1.58E-01 |
| 1.28E-01 | 1.58E-01 |
| 1.29E-01 | 1.58E-01 |
| 1.29E-01 | 1.58E-01 |
| 1.30E-01 | 1.58E-01 |
| 1.31E-01 | 1.58E-01 |
| 1.32E-01 | 1.58E-01 |
| 1.32E-01 | 1.58E-01 |
| 1.33E-01 | 1.58E-01 |
| 1.34E-01 | 1.58E-01 |
| 1.35E-01 | 1.58E-01 |
| 1.36E-01 | 1.58E-01 |
| 1.36E-01 | 1.58E-01 |
| 1.37E-01 | 1.58E-01 |
| 1.38E-01 | 1.58E-01 |
| 1.39E-01 | 1.58E-01 |
| 1.39E-01 | 1.58E-01 |
| 1.40E-01 | 1.58E-01 |
| 1.41E-01 | 1.58E-01 |
| 1.42E-01 | 1.58E-01 |
| 1.43E-01 | 1.58E-01 |
| 1.43E-01 | 1.58E-01 |
| 1.44E-01 | 1.58E-01 |
| 1.45E-01 | 1.58E-01 |
| 1.46E-01 | 1.58E-01 |
| 1.46E-01 | 1.58E-01 |
| 1.47E-01 | 1.58E-01 |
| 1.48E-01 | 1.58E-01 |
| 1.49E-01 | 1.58E-01 |
| 1.49E-01 | 1.58E-01 |
| 1.50E-01 | 1.58E-01 |
| 1.51E-01 | 1.58E-01 |
| 1.52E-01 | 1.58E-01 |
| 1.53E-01 | 1.58E-01 |
| 1.53E-01 | 1.58E-01 |
| 1.54E-01 | 1.58E-01 |
| 1.55E-01 | 1.58E-01 |
| 1.56E-01 | 1.58E-01 |
| 1.56E-01 | 1.58E-01 |
| 1.57E-01 | 1.58E-01 |
| 1.58E-01 | 1.58E-01 |
| 1.59E-01 | 1.58E-01 |
| 1.60E-01 | 1.58E-01 |
| 1.60E-01 | 1.58E-01 |
| 1.61E-01 | 1.58E-01 |
| 1.62E-01 | 1.58E-01 |
| 1.63E-01 | 1.58E-01 |
| 1.63E-01 | 1.58E-01 |
| 1.64E-01 | 1.58E-01 |
| 1.65E-01 | 1.58E-01 |
| 1.66E-01 | 1.58E-01 |
| 1.67E-01 | 1.58E-01 |
| 1.67E-01 | 1.58E-01 |
| 1.68E-01 | 1.58E-01 |
| 1.69E-01 | 1.58E-01 |
| 1.70E-01 | 1.58E-01 |
| 1.70E-01 | 1.58E-01 |
| 1.71E-01 | 1.58E-01 |
| 1.72E-01 | 1.58E-01 |
| 1.73E-01 | 1.58E-01 |
| 1.74E-01 | 1.58E-01 |
| 1.74E-01 | 1.58E-01 |
| 1.75E-01 | 1.58E-01 |
| 1.76E-01 | 1.58E-01 |
| 1.77E-01 | 1.58E-01 |
| 1.77E-01 | 1.58E-01 |
| 1.78E-01 | 1.58E-01 |
| 1.79E-01 | 1.58E-01 |
| 1.80E-01 | 1.58E-01 |
| 1.81E-01 | 1.58E-01 |
| 1.81E-01 | 1.58E-01 |
| 1.82E-01 | 1.58E-01 |
| 1.83E-01 | 1.58E-01 |
| 1.84E-01 | 1.58E-01 |
| 1.84E-01 | 1.58E-01 |
| 1.85E-01 | 1.58E-01 |
| 1.86E-01 | 1.58E-01 |
| 1.87E-01 | 1.58E-01 |
| 1.88E-01 | 1.58E-01 |
| 1.88E-01 | 1.58E-01 |
| 1.89E-01 | 1.58E-01 |
| 1.90E-01 | 1.58E-01 |
| 1.91E-01 | 1.58E-01 |
| 1.91E-01 | 1.58E-01 |
| 1.92E-01 | 1.58E-01 |
| 1.93E-01 | 1.58E-01 |
| 1.94E-01 | 1.58E-01 |
| 1.95E-01 | 1.58E-01 |
| 1.95E-01 | 1.58E-01 |
| 1.96E-01 | 1.58E-01 |
| 1.97E-01 | 1.58E-01 |
| 1.98E-01 | 1.58E-01 |
| 1.98E-01 | 1.58E-01 |
| 1.01E-01 | 1.56E-01 |
| 1.01E-01 | 1.82E-01 |
| 1.01E-01 | 1.79E-01 |
| 1.01E-01 | 1.77E-01 |
| 1.02E-01 | 1.74E-01 |
| 1.02E-01 | 1.72E-01 |
| 1.02E-01 | 1.69E-01 |
| 1.02E-01 | 1.67E-01 |
| 1.02E-01 | 1.64E-01 |
| 1.02E-01 | 1.62E-01 |
| 1.02E-01 | 1.59E-01 |
| 1.02E-01 | 1.51E-01 |
| 1.02E-01 | 1.54E-01 |
| 1.02E-01 | 1.56E-01 |
| 1.01E-01 | 1.50E-01 |
| 1.01E-01 | 1.53E-01 |

**Table C**

MPPT+tracking Pm=[0.105 0.603 0.589 0.812 1.232 2.001 2.432 3.023 3.422 3.714 4.020 4.359 5.251 5.357 6.406 7.546 8.431 9.545 12.443 13.265 14.124 15.307 15.359 16.225 16.325 17.577 18.861 19.572 20.251 20.705 21.411 23.121 22.934 24.301 25.701 27.101 28.471 29.914 30.451 32.323 33.341 35.356 34.451 37.704 38.957 40.575 41.554 42.232 42.652 44.971 45.177 46.530 47.456 49.742 49.595 49.585 50.288 50.602 51.745 52.4631 53.775 53.605 54.440 55.983 56.352 56.747 56.704 57.452 58.466 58.307 59.427 59.385 60.107 58.882 59.120 60.321 58.353 58.920 58.901 57.927 58.334 57.701 58.428 58.750 55.159 55.887 54.136 53.425 52.751 53.424 52.472 50.614 51.539 51.545 50.604 49.851 46.989 46.474 45.652 45.424 44.941 43.925 42.805 42.324 40.852 41.540 38.984 39.327 37.472 34.511 31.525 29.381 27.557 26.434 25.251 24.302 21.523 17.384 16.677 14.462 10.044 5.384 3.727 3.331 1.628 1.461 1.542 1.021 0.956 0.725 0.321 0.105];

Tracking Pd=[0.101 0.601 0.575 0.825 1.158 2.232 2.424 3.332 3.268 3.902 4.153 4.224 4.825 4.978 6.225 6.545 8.3370 10.204 11.749 12.972 14.254 14.535 15.280 15.891 16.298 17.306 18.414 18.451 18.787 19.925 20.405 20.625 20.399 22.357 23.545 24.333 24.714 25.465 26.301 27.364 27.848 28.520 26.951 29.234 30.645 32.548 33.256 33.824 35.347 36.558 37.481 38.121 39.464 39.964 40.220 41.315 42.534 43.037 43.804 44.422 45.495 46.304 47.441 48.505 49.251 49.424 49.837 49.980 50.411 50.274 50.833 51.318 52.363 52.375 51.628 50.418 50.257 51.614 50.650 50.313 50.251 49.567 49.101 50.425 49.514 49.158 47.2140 47.441 46.452 45.501 44.582 45.924 44.833 45.354 43.3140 43.623 41.208 40.954 40.240 40.244 39.311 38.554 37.927 37.831 36.377 36.306 34.983 34.397 33.824 31.604 30.201 28.375 26.552 26.027 24.396 23.263 20.554 17.208 16.453 13.334 10.014 5.136 3.725 3.382 1.527 1.233 1.045 1.525 1.277 0.712 0.207 0.101];

Fixed installation Px=[0.100 0.113 0.158 0.435 0.325 0.576 0.789 0.935 1.105 1.324 1.552 1.946 2.221 2.738 2.554 3.670 3.392 3.754 4.031 4.405 5.942 5.380 6.569 6.859 7.508 8.921 8.768 9.381 9.650 10.551 12.258 13.237 13.551 13.354 14.305 15.304 15.655 16.981 18.051 19.362 20.464 22.370 21.336 21.901 23.946 25.264 25.975 27.386 27.535 28.943 30.210 30.982 31.802 34.581 35.765 36.341 37.358 38.494 40.435 41.520 42.337 43.424 44.241 44.724 46.652 48.127 47.611 48.324 48.674 48.991 50.480 49.937 50.564 51.354 50.925 49.304 49.336 49.392 48.282 48.207 47.892 46.435 46.201 47.425 45.633 44.782 44.421 43.571 42.405 41.314 40.337 38.550 37.741 37.698 35.407 34.543 33.908 31.699 29.837 29.922 28.685 28.324 24.551 23.297 21.964 22.629 21.736 19.638 17.915 15.873 14.408 13.550 12.927 11.735 10.902 9.438 8.872 7.393 6.556 5.727 4.533 2.274 2.118 1.751 1.041 0.883 0.422 0.351 0.324 0.303 0.251 0.099];

**Table D**

MPPT+tracking Pm=[0.0417 0.2917 0.4583 0.5833 0.8333 1.0417 1.2500 1.0000 1.4167 1.7083 1.8333 2.0417 2.2083 2.5833 2.6250 3.5000 4.0000 5.2083 5.4583 5.5417 6.2500 8.5333 10.3250 10.6833 10.6833 10.8917 10.7417 11.3583 12.6083 14.4917 15.7833 16.6750 17.2667 18.0583 18.8917 19.3500 18.9333 20.1000 20.2250 20.6417 20.9333 21.1583 21.4750 21.6833 21.6900 21.7667 21.5583 21.8917 21.9333 21.8917 21.6833 22.1333 22.4917 22.7417 23.3250 23.5750 23.6583 23.3250 24.0750 24.4583 24.4917 24.8667 24.6833 23.9750 23.8500 23.9750 24.3667 23.9750 24.2250 24.7667 24.8917 24.6833 25.1000 24.3500 23.9500 23.8917 23.7967 23.6417 23.6583 23.9333 23.1000 22.6833 22.6833 22.6833 21.9750 21.5167 21.9750 21.0167 20.5000 19.7083 19.9583 19.7083 18.9167 18.6250 18.5417 17.5083 17.3917 16.4750 15.3250 13.6000 13.4750 13.1417 12.3083 11.5167 11.1167 10.5750 10.6417 10.2000 8.9750 8.9167 7.2083 5.5833 4.7500 5.0000 3.9167 3.5017 3.2590 2.8937 2.2193 2.1875 2.0853 1.9583 1.7917 1.7917 1.5000 1.3750 1.0417 0.6667 0.5000 0.2917 0.0833 0.0417];

Tracking Pd=[ 0.0417 0.2500 0.4583 0.5833 0.7917 1.0417 1.2500 1.0417 1.4167 1.6250 1.7500 1.9167 2.1250 2.5000 2.5417 3.1667 3.8750 5.0000 5.1250 5.3333 5.9583 8.3333 9.6250 10.1250 10.1667 10.3333 10.0000 10.5417 10.9583 12.1667 14.2917 15.1250 15.8333 16.5000 17.2083 17.4583 17.6250 17.1667 17.9167 18.0417 18.5000 18.5417 18.7500 18.9167 19.3750 19.5833 19.7083 19.7500 19.9167 19.7917 20.0833 19.6250 20.1667 20.4167 20.5417 20.7083 20.9167 20.5417 20.7500 20.9167 21.2083 21.2917 21.4167 21.0000 21.0417 20.9167 21.6250 21.3750 21.6250 21.5417 21.5000 22.1667 21.9167 22.2083 21.7917 21.7918 21.7500 21.6250 21.5417 21.7083 21.4167 20.6167 20.5417 20.0933 19.6833 19.4667 19.7201 19.1496 18.4583 18.3750 17.5833 17.0000 16.3333 14.7500 14.0417 13.9167 12.8750 12.5000 11.1667 10.0833 9.8750 9.7917 9.7500 9.4667 9.2917 9.2250 9.2500 8.5083 8.2000 8.0333 7.1250 5.5417 4.7083 4.8750 3.8750 3.4583 3.1667 2.8333 2.1250 2.1250 2.0833 1.8333 1.7500 1.7500 1.4167 1.3333 0.9167 0.6250 0.3333 0.2500 0.0823 0.0407];

Fixed installation Px=[ 0.0417 0.0417 0.0833 0.0833 0.1250 0.2500 0.3333 0.4167 0.4167 0.5417 0.7500 0.9167 1.1667 1.5417 1.4167 1.7500 1.8333 2.0417 2.1667 2.2917 2.7083 3.4167 4.8333 5.2083 5.3750 5.5417 5.6250 6.2500 6.7917 7.2500 7.6667 8.6667 9.3750 10.1250 10.5833 11.4167 12.6667 13.1667 14.2917 14.7083 15.5000 15.6667 15.7917 15.9583 16.0417 16.2500 16.7083 17.1250 17.3333 17.5417 17.8750 17.9167 17.6250 18.2000 18.5750 18.9750 19.4900 19.6750 19.8750 19.8750 20.5800 20.6833 20.6333 20.5417 20.3750 20.1833 20.2983 19.9167 20.3917 20.2250 19.8500 20.1167 19.8500 19.1833 18.6000 18.5167 18.5167 18.1000 17.9583 17.6583 16.8733 16.5167 16.1000 15.7667 15.2000 14.9967 14.3250 13.6000 11.9750 11.8917 11.6167 10.8250 10.2833 10.2625 9.9375 9.5417 8.5750 8.2833 8.6583 8.2000 8.2000 7.8333 8.0417 7.9333 6.8500 6.6833 6.8500 6.5167 5.3250 4.4083 3.6583 3.6375 3.2417 3.2833 2.4500 2.2667 1.8917 1.4750 1.3500 1.5167 1.3500 0.9167 0.8917 0.3600 0.2808 0.2917 0.1767 0.0933 0.0933 0.0933 0.0517 0.0208];

**Table E**

MPPT+tracking Pm=[ 0.0240 0.0360 0.0360 0.0960 0.1200 0.3000 0.4800 0.7200 0.9600 1.2000 1.6800 1.9200 2.2072 2.5477 2.7212 3.2589 3.3743 4.5843 4.4200 5.2000 5.6940 6.6226 6.6316 7.1944 8.3021 8.9548 9.5940 11.5216 12.5832 15.7741 15.9690 19.3162 20.1432 20.6517 20.1494 20.1436 20.9044 20.7493 19.8712 20.2202 19.6620 19.0890 19.2518 19.2773 18.3804 18.7375 18.4848 17.8442 17.7426 17.9217 17.2597 16.7956 18.0810 18.0591 17.6136 17.4240 18.5376 17.7744 18.1702 18.0327 17.7909 19.1500 19.3000 18.3900 19.5320 19.4412 19.1520 19.3000 19.3158 19.3784 19.2072 19.3980 20.3626 20.0560 19.1568 19.1012 19.3980 19.8880 20.3285 20.4573 19.5590 18.9210 18.0480 18.0198 17.5686 15.6529 15.4177 14.7000 14.6592 14.9240 14.5600 13.6500 14.0448 11.5500 11.6250 11.2413 11.3261 13.3000 13.0940 12.7416 12.3604 11.8923 11.6676 11.2732 10.8966 10.3162 10.5600 9.9761 8.9191 9.0294 7.5719 5.3754 4.7811 4.5922 4.0943 3.7141 3.4525 3.2614 2.8100 2.4784 2.0812 2.1024 1.8382 1.8876 1.3650 1.3596 1.0560 0.7260 0.8580 0.9240 0.4752 0.3960 0.2640];

Tracking Pd=[ 0.0120 0.0240 0.0480 0.0600 0.0840 0.1080 0.1200 0.3000 0.4440 0.5400 0.8280 0.8400 1.1318 1.3442 1.4957 1.5973 1.7957 1.9859 2.3568 2.5452 2.9618 3.4498 4.0018 4.2728 4.7306 5.4952 5.6330 6.2284 7.0198 7.0384 7.8778 9.1522 9.5374 11.2237 12.3827 12.9996 13.5375 14.4144 14.8571 15.1187 15.5480 15.4611 15.0146 14.7548 14.2444 14.2295 13.8434 13.5090 13.2150 12.9891 12.7389 12.4085 12.3629 12.7097 12.9443 12.6392 13.6993 13.4845 13.5903 13.3200 13.4724 14.0765 14.0521 13.9546 14.3406 14.2752 14.4592 13.6694 14.4354 15.3209 15.1711 15.1277 14.8800 15.0486 14.7009 14.8502 15.3023 15.0688 15.3935 15.8119 15.7027 15.5467 14.0754 13.1330 13.2105 12.6586 12.7652 12.2826 11.9509 11.8694 11.6084 11.3164 10.6787 8.3240 8.6073 8.4540 8.4916 10.2393 9.5190 9.5807 9.8042 9.6064 9.5658 7.8586 6.7330 6.5960 6.1352 5.5202 4.7087 4.4889 3.7696 3.5192 3.2365 3.1011 2.9843 2.6406 2.2962 1.8059 1.5785 1.3134 1.1483 0.7878 0.3860 0.2720 0.1965 0.1672 0.1310 0.1279 0.0917 0.0786 0.0655 0.0393 0.0131];

Fixed installation Px=[ 0.0240 0.0360 0.0480 0.0960 0.1200 0.3000 0.4800 0.7200 0.9600 1.1400 1.5600 1.8000 2.0893 2.4428 2.5979 3.1801 3.3840 4.4634 4.5430 5.0713 5.7203 6.5167 6.5381 7.2435 7.9799 9.0127 9.1621 10.3607 11.4240 12.4267 13.9731 14.4231 17.2385 19.0277 18.3830 18.6326 16.5589 17.2104 16.9425 16.6179 15.9845 15.9758 15.4877 15.1451 15.1046 14.9240 14.3998 13.8635 13.7799 13.8218 13.3458 12.9086 13.3520 13.5207 13.1798 13.2105 14.0790 13.7492 13.9090 13.9590 13.8932 14.7954 14.6365 14.4894 14.6867 14.8551 14.7758 14.2434 15.4980 15.7304 15.6030 16.1647 16.6663 16.4062 15.9039 15.9180 16.2212 16.6663 17.0500 17.2119 16.4641 15.9937 15.3816 15.2490 14.8851 13.2634 13.2396 12.8592 12.9703 13.0302 12.4467 11.6603 12.0329 9.4702 9.4907 9.1692 9.3384 11.7150 11.5119 10.9135 10.4801 10.1824 10.1127 10.0532 9.8343 9.5227 9.7094 8.7116 8.6029 8.8231 7.3797 5.2555 4.6945 4.4392 3.9484 3.5939 3.3341 3.2483 2.7797 2.4416 2.0614 1.9962 1.7952 1.7820 1.2804 1.3200 0.9900 0.6600 0.9240 0.7920 0.5280 0.3960 0.2640];
